# Moonlighting cytosolic function of ACAD9: suppression of TRAF6-mediated osteoclastogenesis and protection against osteoporosis

**DOI:** 10.1038/s41419-026-08626-z

**Published:** 2026-03-26

**Authors:** Mimi Wang, Chao Yuan, Yi Zhang, Mengmeng Peng, Yundie Liu, Ruolin Liu, Zhaode Feng, Zhiwei Yang, Hao Li, Zhongbo Liu, Ying Cheng

**Affiliations:** 1 Key Laboratory of Biomedical Information Engineering of Ministry of Education, Center for Mitochondrial Biology & Medicine, School of Life Science and Technology, Xi’an Jiaotong University, Xi’an, China; 2 Key Laboratory of Shaanxi Province for Craniofacial Precision Medicine Research, Laboratory Center of Stomatology, College of Stomatology, Xi’an Jiaotong University, Xi’an, China; 3 MOE Key Laboratory for Nonequilibrium Synthesis and Modulation of Condensed Matter, School of Physics, Xi’an Jiaotong University, Xi’an, China

**Keywords:** Cell signalling, Post-translational modifications

## Abstract

Acyl-CoA dehydrogenase-9 (ACAD9) is classically known for its role in mitochondrial fatty acid β-oxidation and complex I assembly. Here, we identify ACAD9 deficiency as a clinically relevant risk factor for fragility fractures and reveal a previously unrecognized cytosolic function of ACAD9 in suppressing osteoclast differentiation, thereby protecting against osteoporosis. Mechanistically, while preserving its canonical mitochondrial role in complex I assembly, we find that ACAD9 also facilitates the formation of respiratory chain supercomplexes. Notably, in the cytosol, ACAD9 competitively binds to TRAF6, preventing its interaction with the E2 ubiquitin-conjugating complex UBC13/UEV1A, and thereby blocking K63-linked polyubiquitination and downstream activation of the RANK/TRAF6/TAK1/NFATc1 signaling cascade. Additionally, ACAD9 promotes K48-linked polyubiquitination of TRAF6, leading to its proteasomal degradation. Osteoclast-specific *Acad9* knockout mice exhibit increased osteoclast numbers and decreased bone mass. These findings uncover a novel extramitochondrial function of ACAD9 in regulating osteoclast differentiation and maturation, and offer potential therapeutic insights for targeting osteoclast hyperactivity in osteoporosis.

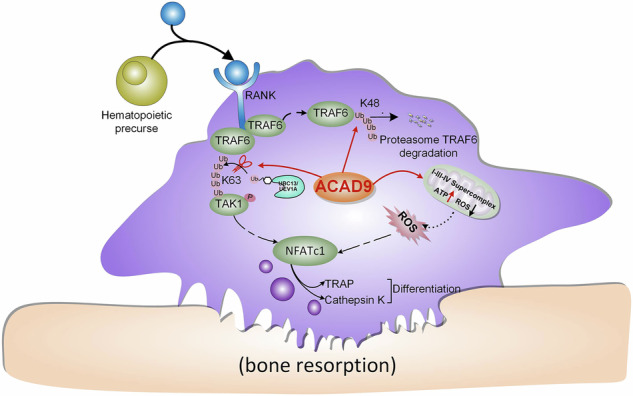

## Introduction

Bone maintains homeostasis through continuous remodeling involving balanced resorption and formation [1, 2]. When osteoclast activity exceeds osteoblast activity capacity, disruption of this balance leads to skeletal disorders like osteoporosis [1, 3]. Osteoclasts, the only cells capable of bone resorption, differentiate from their precursor cells through osteoclastogenesis that depends on RANKL and M-CSF signaling [1–7]. The RANKL-RANK interaction is particularly critical for osteoclast maturation, making this signaling pathway a key therapeutic target for osteoporosis.

Osteocytes and osteoblasts secrete RANKL, which activates TRAF6 through RANK binding. This induces NFATc1 via NF-κB/MAPK pathways or ROS generation. These signals upregulate osteoclast markers (TRAP, Cathepsin K, MMP9) and promote differentiation [8, 9]. In addition, TRAF6 activates Rac1 to stimulate membrane NOX1/2, boosting intracellular ROS. Mitochondria also generate ROS via the respiratory chain complex (I–V) during oxidative phosphorylation [10–12]. These complexes can form respiratory chain supercomplexes (SCs), CI₁ + CIII₂ + CIV_n_ SCs enhance ATP synthesis while reducing ROS [13, 14], and the assembly factors of SCs ensure SC stability [15, 16]. Intriguingly, mature osteoclasts exhibit a characteristic metabolic shift with decreased ATP and elevated ROS levels, indicating that SCs remodeling may play a pivotal role in osteoclastogenesis [17, 18]. Notably, the identification of key mitochondrial assembly factors regulating these dynamic structural changes could provide novel therapeutic targets for bone metabolic disorders, although their precise roles in osteoclast differentiation remain to be elucidated.

ACAD9 is a multifunctional protein with well-established roles in mitochondrial fatty acid β-oxidation and respiratory chain complex I assembly through its interactions with NDUFAF1 and ECSIT [19, 20]. Beyond these canonical mitochondrial functions, emerging evidence suggests that ACAD9 may play additional roles in cytoplasmic signaling pathways during osteoclast differentiation. This potential dual functionality is particularly intriguing given the critical metabolic reprogramming that occurs during osteoclastogenesis, characterized by elevated ROS production and decreased ATP levels [17, 18]. Structural analysis reveals that ACAD9 contains a highly conserved TRAF6-binding motif (77-Pro-Leu-Glu-Phe-Phe-82) that perfectly matches the established consensus sequence (Pro-X-Glu-X-X-aromatic/acidic) for TRAF6 interaction [21]. This motif shows remarkable similarity to the TRAF6-binding domain in ECSIT, a known regulator of NF-κB signaling [22]. This strongly suggests that ACAD9 may similarly interact with TRAF6 in the cytoplasm. Studies found that TRAF6 plays a central role in osteoclastogenesis by activating NF-κB and MAPK pathways to induce NFATc1 expression. As an E3 ubiquitin ligase, TRAF6 mediates K63-linked polyubiquitination through its RING domain in complex with UBC13/UEV1A [23, 24]. These ubiquitin chains serve as scaffolds to recruit and activate the TAK1-TAB1-TAB2 complex, initiating downstream signaling [25–27]. Therefore, we speculate that ACAD9 may influence osteoclast differentiation and maturation by regulating TRAF6 activity.

In this study, we uncover the novel dual role of ACAD9 in osteoclastogenesis. ACAD9 has a critical function in mitochondrial respiration via complex I and SCs assembly. Additionally, ACAD9 acts as a TRAF6-interacting regulator of the NF-κB/MAPK pathway. ACAD9 thus emerges as a pivotal checkpoint in bone resorption. Targeting the ACAD9-TRAF6 axis represents a promising dual-mechanism therapeutic strategy for osteoporosis. This approach would simultaneously improve mitochondrial function and suppress excessive osteoclast activation to prevent pathological bone loss.

## Results

### ACAD9 expression is downregulated during osteoclast differentiation and osteoporosis

**Fig. 1:**
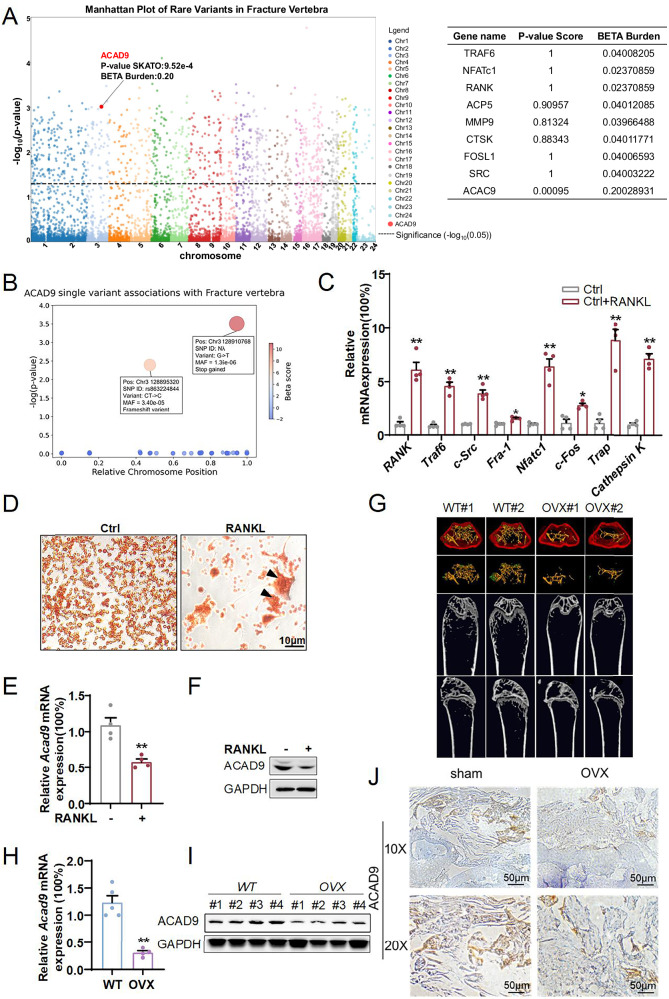
ACAD9 expression is downregulated during osteoclast differentiation and osteoporosis. **A** Manhattan Plot of Rate variant about ACAD9 in Fracture Vertebra. **B** The beta score and *P* value for all single-variant information related to ACAD9. RAW264.7 cells were induced to differentiate for 4 days with or without 100 nM RANKL, (**C**) relative mRNA expression levels of markers of osteoclast differentiation. **D** TRAP staining, (**E**) relative mRNA, and (**F**) protein expression levels of ACAD9. **G** Representative Micro-CT images of the femurs of wild-type and OVX mice. **H** mRNA and (**I**) protein expression levels of ACAD9 were tested in wild-type and OVX mice. **J** Frozen sections were prepared from wild-type and OVX mice and immunostained with an anti-ACAD9 antibody. The beta score and *P* value for all single-variant information related to ACAD9. Data are shown as Mean ± SEM, ∗*p* < 0.05, ∗∗ *p* < 0.01 vs. the Ctrl group. For in vitro experiments, data represent at least three independent experiments. For in vivo studies, *n* = 4–5 mice per group were used.

Osteoporosis is characterized by progressive bone loss and increased fracture risk. To investigate whether ACAD9 is associated with osteoporosis in humans, we analyzed rare variant data from the Genebass browser (UK Biobank cohort). Gene-level SKAT-O analysis revealed a significant association between ACAD9 pLoF variants and vertebral fractures (*p* = 9.5 × 10⁻⁴, burden beta = 0.20) (Fig. 1A). Two high-impact mutations were particularly notable: a G→T substitution (Chr3:128910768, beta = 10.36, *p* = 3.14 × 10⁻⁴) introducing a premature stop codon, and a CT→C frameshift (Chr3:128895320, beta = 6.73, *p* = 4.01 × 10^−^^3^) causing aberrant translation (Fig. 1B). These ultra-rare variants (MAF ~10⁻⁶–10⁻⁵) affect only a small subset of individuals. While single-variant effect sizes are unstable for such rare alleles, consistent positive directionality across independent pLoF variants supports a gene-level effect, suggesting ACAD9 deficiency may contribute to fracture susceptibility. And more, we utilized an in vitro model with RAW264.7 cells induced by 100 nM RANKL to simulate osteoclast differentiation. It showed significant upregulation of mRNA levels of osteoclastic regulator factors such as *RANK*, *Traf6*, *c-Src*, and *Fra-1*, as well as transcription factors *Nfatc1* and *c-Fos*, along with markers *Trap* and *Cathepsin K* (Fig. 1C). TRAP staining revealed extensive cell fusion and formation of large multinucleated cells after 4 days of 100 nM RANKL treatment (Fig. 1D). These data indicate robust differentiation of RAW264.7 cells. We found that ACAD9 mRNA and protein expression levels significantly decreased in differentiated cells (Fig. 1E, F). Subsequently, we established an in vivo osteoporosis model using ovariectomized (OVX) mice to simulate estrogen deficiency-induced osteoporosis. Micro-computed tomography(μCT) analysis demonstrated a significant decrease in bone volume per tissue volume in OVX mice (Fig. 1G). Consistently, ACAD9 mRNA and protein levels were significantly reduced in the bones of OVX mice (Fig.1H, I). Immunohistochemistry staining results further confirmed reduced ACAD9 levels in OVX mice (Fig.1J). Together, these data suggested a potential role for ACAD9 in osteoclast differentiation and osteoporosis.

### Downregulation of ACAD9 promotes osteoclast differentiation and maturation

**Fig. 2:**
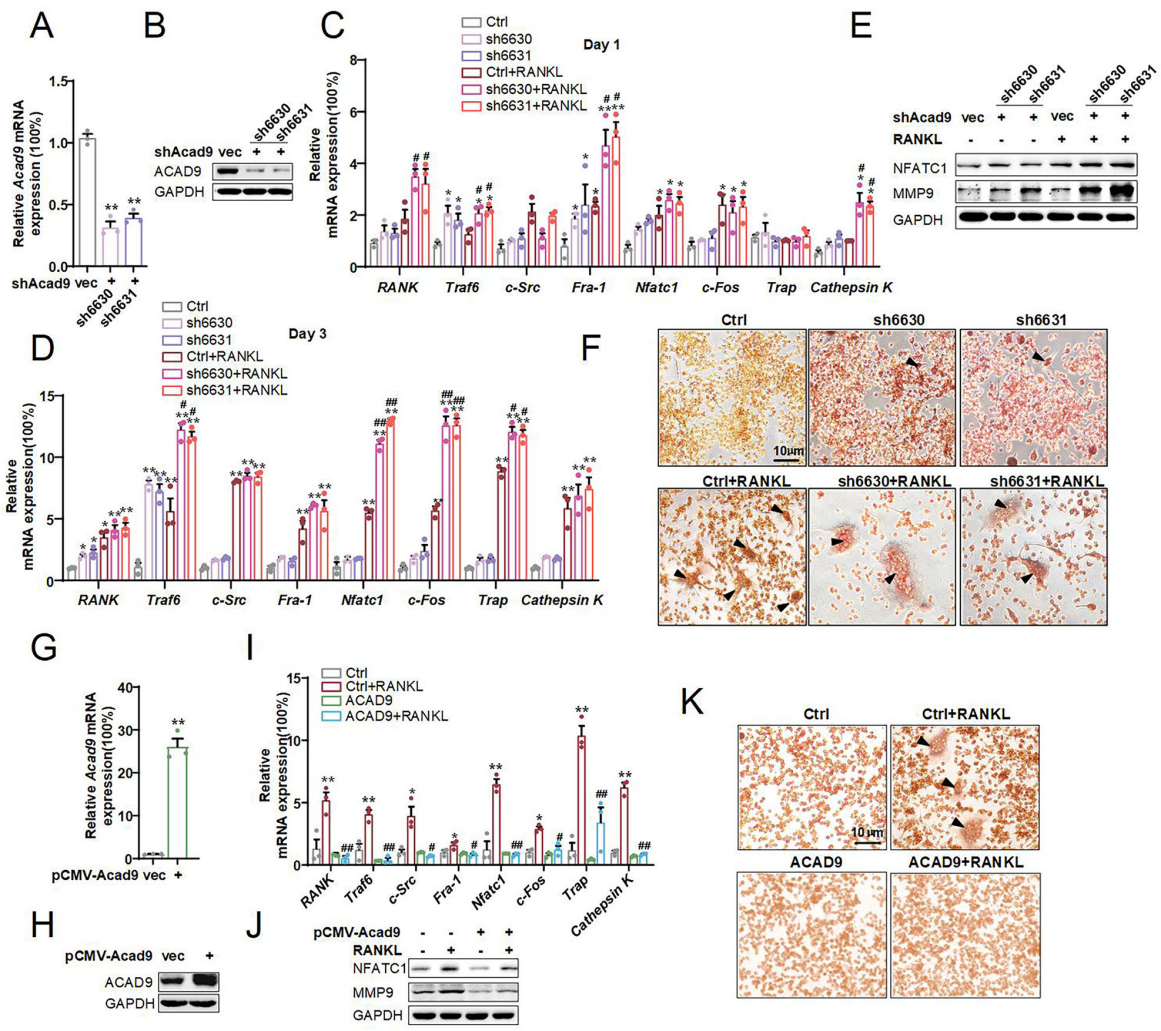
Downregulation of ACAD9 promotes osteoclast differentiation and maturation. **A** mRNA and (**B**) protein expression levels of ACAD9 were detected by RT-PCR and western blotting in *Acad9* stable knockdown cells. **C**, **D** The relative mRNA levels of markers of osteoclasts were detected by RT-PCR after induced differentiation for 1 day and 3 days. **E** The protein expression levels of markers of osteoclast differentiation were detected by western blotting, and (**F**) cells were performed TRAP staining after induced differentiation for 4 days. **G** mRNA and (**H**) protein expression levels of ACAD9 were detected by RT-PCR and western blotting in *Acad9* stable overexpressing cells. **I** The relative mRNA levels of markers of osteoclasts were detected by RT-PCR after induced differentiation for 4 days. **J** The protein expression levels of markers of osteoclast differentiation were detected by western blotting, and (**K**) cells were performed TRAP staining after induced differentiation for 4 days. Data are shown as Mean ± SEM, ∗*p* < 0.05, ∗∗ *p* < 0.01 vs. Ctrl group; # *p* < 0.05, ## *p* < 0.01 vs. Ctrl+RANKL group. For in vitro experiments, data represent at least three independent experiments.

To investigate the role of ACAD9 in osteoclast differentiation and maturation, we stably knocked down *Acad9* using two specific shRNAs (Fig. 2A, B). mRNA levels of *RANK*, *Traf6, Fra-1, Nfatc1*, and *Cathepsin K* were significantly elevated after just 1 day of RANKL treatment (Fig. 2C). Additionally, a few fusion and multinucleated cells were found in *Acad9* knockdown cells even without RANKL treatment (Fig. 2F, upper). These indicate a predisposition to differentiation following the inhibition of ACAD9 expression. Notably, compared to control cells, mRNA levels of *Traf6*, *Nfatc1*, *c-Fos*, and *Trap* were dramatically increased in *Acad9* knockdown cells after 3 days of RANKL treatment (Fig. 2D). Additionally, there was a significant increase in the protein expression levels of NFATc1 and MMP9 in *Acad9* knockdown cells after 4 days of RANKL treatment (Fig. 2E). Furthermore, the degree of cell fusion and the size of multinucleated cells were significantly larger in *Acad9* knockdown cells under RANKL treatment (Fig. 2F, lower). These findings strongly indicate that downregulated expression of ACAD9 promotes osteoclast differentiation. Furthermore, we stably overexpressed *Acad9* using pCMV-*Acad9* plasmids. It showed that overexpression of ACAD9 inhibits osteoclast differentiation (Fig. 2G–K). Taken together, these data highlight ACAD9 as a negative regulator in osteoclast differentiation.

### ACAD9 decreases ROS production by promoting the assembly of mitochondrial complex I and SCs, thereby inhibiting osteoclast differentiation

RANKL-induced ROS production is an important regulatory factor in osteoporosis and fractures [9]. During osteoclast differentiation, we observed a significant increase in ROS levels. When cells were treated with NAC, a known ROS scavenger, ROS levels dramatically decreased (Supplementary Fig. S1A). Furthermore, NAC significantly inhibited osteoclast differentiation. This was evidenced by reduced expression of differentiation markers and decreased cell fusion with fewer large multinucleated cells (Supplementary Fig. S1B–D). These results indicate that ROS plays an important role in maintaining osteoclast differentiation and osteoporosis.

**Fig. 3:**
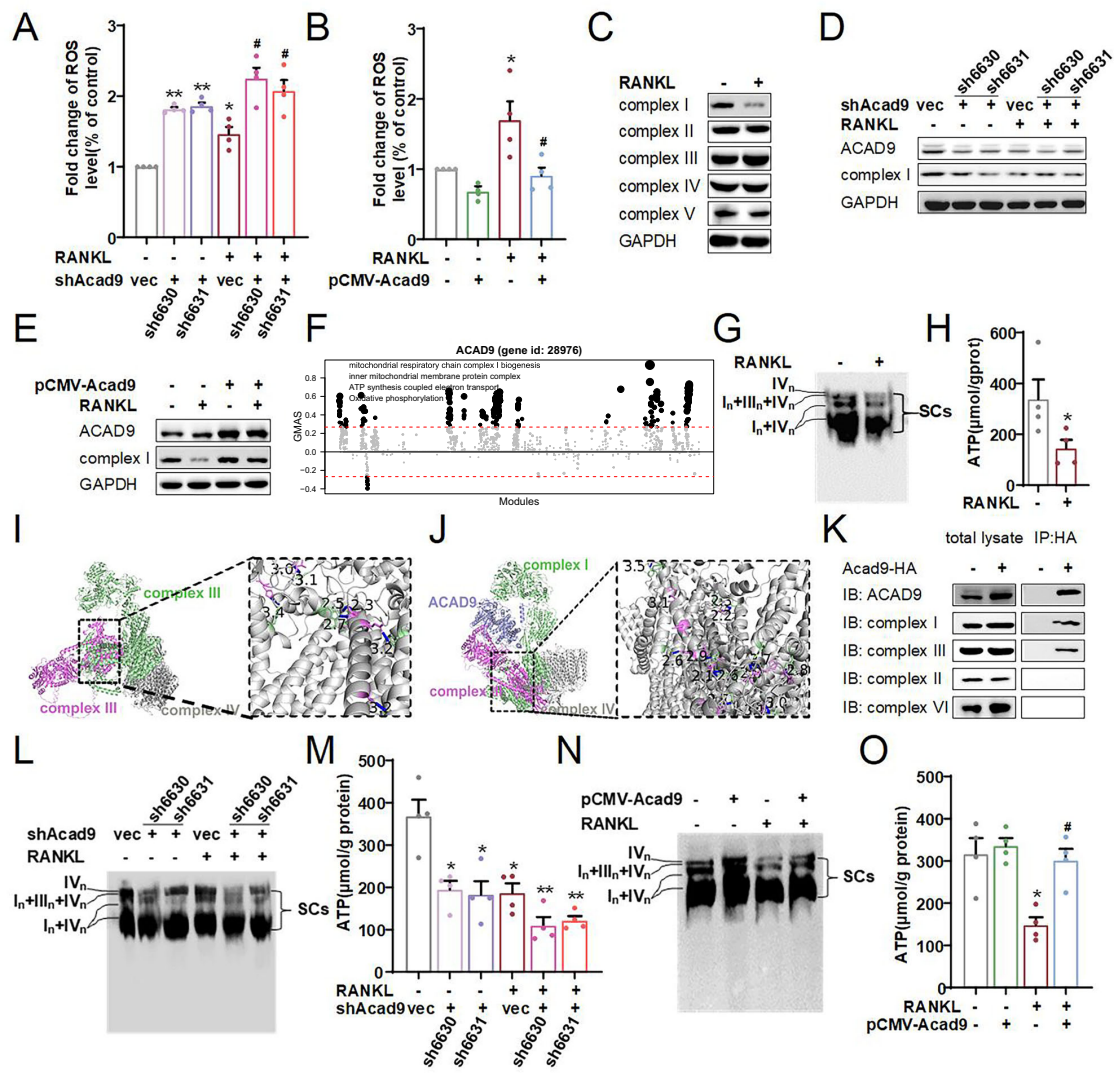
ACAD9 decreases ROS production by promoting the assembling of mitochondrial complex I and SCs, thereby inhibiting osteoclast differentiation. ROS levels were detected using DCFH2-DA in the cells. **A** ROS levels from cells that stably knockdown ACAD9 after induced 4 days. **B** ROS levels from cells that overexpressed *Acad9* after induced 4 days. **C** Mitochondrial complex I–V were detected by western blotting from RAW264.7 cells. Mitochondrial complex subunit I was detected from cells that (**D**) stably knockdown *Acad9* or (**E**) overexpressed *Acad9*. **F** G-MAD revealed the potential role of ACAD9 in bone and bone marrow tissues. **G**, **L**, **N** Immunoblots for SCs by BN-PAGE and immunoblot with NDUFA9 (subunit of Complex I) and (**H**), (**M**), (**O**) concentrations of ATP were detected by assay kit in the indicated RAW264.7 cells. AlphaFold3 predicted the interaction of complex subunits I/II/III **I** in the absence of ACAD9, or (**J**) in the presence of ACAD9, as shown. **K** Co-IP analysis on the interaction between ACAD9 and complex subunits of I, II, III, and IV. Data are shown as Mean ± SEM, ∗*p* < 0.05, ∗∗ *p* < 0.01 vs. Ctrl group; # *p* < 0.05, ##*p* < 0.01 vs. Ctrl+RANKL group. For in vitro experiments, data represent at least three independent experiments.

Mitochondria are considered the primary sites of endogenous ROS production. Therefore, we investigated whether ACAD9 influences osteoclast differentiation by regulating the production of ROS. First, we measured ROS levels and found that ROS was significantly elevated in stable knockdown ACAD9 cells, regardless of RANKL treatment or not, when compared to normal cells (Fig. 3A). Conversely, we found that ROS levels were significantly decreased in stable overexpressing *Acad9* cells under RANKL treatment when compared to normal cells (Fig. 3B). To investigate the impact of ACAD9 on the production of ROS, we conducted a Gene Ontology analysis about ACAD9 and markers of osteoclast differentiation, revealing a strong correlation with oxidative stress, mitochondrial inner membrane composition, and mitochondrial function (Supplementary Fig. S2). Therefore, we focused on the protein expression levels of mitochondrial complexes I, II, III, IV, and V, which are important for the generation of ATP and ROS. We observed a significant decrease specifically in the expression of complex I in RANKL-induced differentiated cells (Fig. 3C). Subsequently, we observed a significant decrease in the expression levels of complex I in stable knockdown *Acad9* cells, both with and without RANKL treatment (Fig. 3D). We also found that the expression levels of complex I in stable overexpressing *Acad9* cells were restored compared to those in normal RANKL-treated cells (Fig. 3E). These findings indicated that ACAD9 influences intracellular ROS production by modulating mitochondrial complex I assembly.

To investigate the new function of ACAD9 beyond its role in the assembly of complex I, we utilized G-MAD to identify novel functions [28]. We applied the GeneBridge tool to explore modules associated with *Acad9* in bone and bone marrow. We identified that ACAD9 not only regulates mitochondrial membrane protein complex and ATP synthesis coupled electron transport, but also influences oxidative phosphorylation (Fig. 3F). Studies have demonstrated that the mitochondrial electron transport chain complexes can assemble into SCs through different combinations, thereby enhancing electron transport efficiency and ATP production, and reducing ROS generation. Specifically, the configuration of SCs composed of CI_1_ + CIII_2_ + CIV_2_ has been documented [29]. Therefore, we speculated that ACAD9 not only regulates the assembly of mitochondrial complex I but may also promote the assembly of SCs, thereby influencing ROS generation and ATP production. We found that the protein levels of SCs significantly decreased in RANKL-treated cells, correlating with a notable decrease in ATP content (Fig. 3G, H). Subsequently, we predicted that ACAD9 interacts with both mitochondrial complexes I and III, promoting tighter binding between their subunits within the SCs by AlphaFold3(Fig. 3I, J). Furthermore, we verified that ACAD9 does bind to mitochondrial complexes I and III subunits (Fig. 3K). Moreover, compared to normal cells, we found that protein levels of the SCs and ATP content significantly decreased in stable *Acad9* knockdown cells both with and without RANKL treatment (Fig. 3L, M), and significantly increased in *Acad9* overexpressing cells (Fig. 3N, O). These results indicate that ACAD9 plays a critical role in reducing intracellular ROS levels by promoting the assembly of mitochondrial complex I and SCs, ultimately inhibiting osteoclast differentiation.

Most interestingly, if ACAD9 regulates osteoclast differentiation solely through adjusting the generation of ROS, then adding NAC to clear the intracellular ROS should completely suppress the osteoclast differentiation in stable knockdown *Acad9* cells. We added NAC into the stable knockdown *Acad9* cells and found that clearing ROS could only suppress expression levels of some markers of osteoclast differentiation, such as *Trap* and *Cathepsin K* (Supplementary Fig. S1E). However, other markers of differentiation and those proteins directly related to bone resorption marginally recovered to normal levels (Supplementary Fig. S1E, F). Moreover, TRAP staining data further demonstrated that the morphology of cell fusion and formation of large multinucleated cells did not show significant improvement after NAC treatment (supplementary Fig. S2G).

Altogether, these results about ROS suggested that ACAD9 regulates osteoclast differentiation partially by regulating intracellular ROS production; however, ACAD9-complex/SCs -ROS may not be the only signaling pathway involved in the regulation of osteoclast differentiation. ACAD9 certainly influences other molecules or signaling pathways, beyond the mitochondrial complex and SCs, thereby regulating osteoclast differentiation.

### ACAD9 inhibits TRAF6-mediated activation of NOX1/2 and MAPK/NF-κB signaling pathways during osteoclastogenesis

**Fig. 4:**
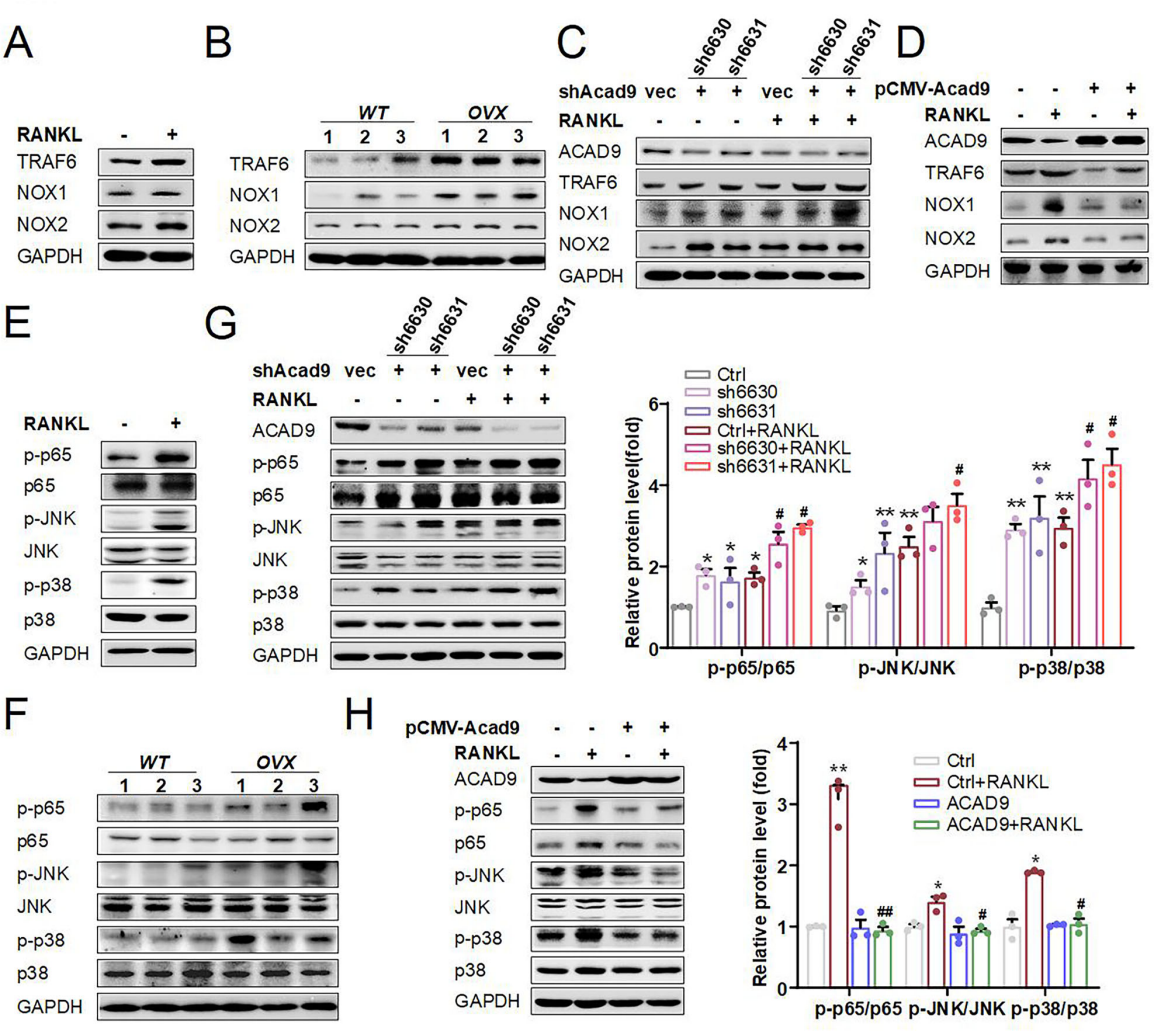
ACAD9 inhibits TRAF6-mediated activation of NOX1/2 and MAPK/NF-κB signaling pathways during osteoclastogenesis. The protein expression levels of downstream of TRAF6 were detected by western blotting (**A**, **C**, **D**, **E**, **G**, **H**) in indicated RAW264.7 cells (**B**, **F**) or from femurs of wild-type and OVX mice (*n* = 3 mice per group). **G**, **H** (right) Relative protein phosphorylation levels were quantified. Data are shown as Mean ± SEM, ∗*p* < 0.05, ∗∗ *p* < 0.01 vs. Ctrl group; # *p* < 0.05, ##*p* < 0.01 vs. Ctrl+RANKL group.

As mentioned above, ACAD9 may influence other factors that affect osteoclast differentiation. Studies have shown that when RANKL binds to RANK, it recruits its downstream target molecule TRAF6, leading to TRAF6 activation. Activated TRAF6 plays a dual role: it regulates Rac1/NOX signaling, promoting intracellular ROS production; it promotes osteoclast differentiation by activating the MAPK and NF-κB signaling pathways [9, 30, 31]. Therefore, we assessed the protein expression levels of TRAF6 and NOX1/2 both in vitro and in vivo. As expected, they were significantly elevated in RANKL-treated cells and OVX mice (Fig. 4A, B). Moreover, compared to normal cells, knockdown of *Acad9* resulted in an obviously increased expression of TRAF6 and NOX1/2, while overexpressing *Acad9* led to a significant decrease under RANKL treatment (Fig. 4C, D). These results suggest that ACAD9 regulates TRAF6/NOXs signaling pathways and then impacts osteoclastogenesis. Further, we assessed the activation status of NF-κB/MAPK. As expected, the phosphorylation levels of p65, JNK, and p38 were significantly elevated in cells under RANKL treatment and in OVX mice (Fig. 4E, F). Consistently, the phosphorylation levels of p65, JNK, and p38 were significantly increased in *Acad9* knockdown cells compared to normal cells, while they were dramatically decreased in A*cad9* overexpression cells under RANKL treatment (Fig. 4G, H). Notably, knockdown of *Acad9* can activate NF-κB/MAPK signaling even in the absence of RANKL treatment (Fig. 4G). These results suggest that ACAD9 plays a vital role in regulating TRAF6/NF-κB/MAPK signaling pathways.

### ACAD9 inhibits RANKL-induced osteoclast differentiation by regulating TRAF6 ubiquitination, modification, and degradation

**Fig. 5:**
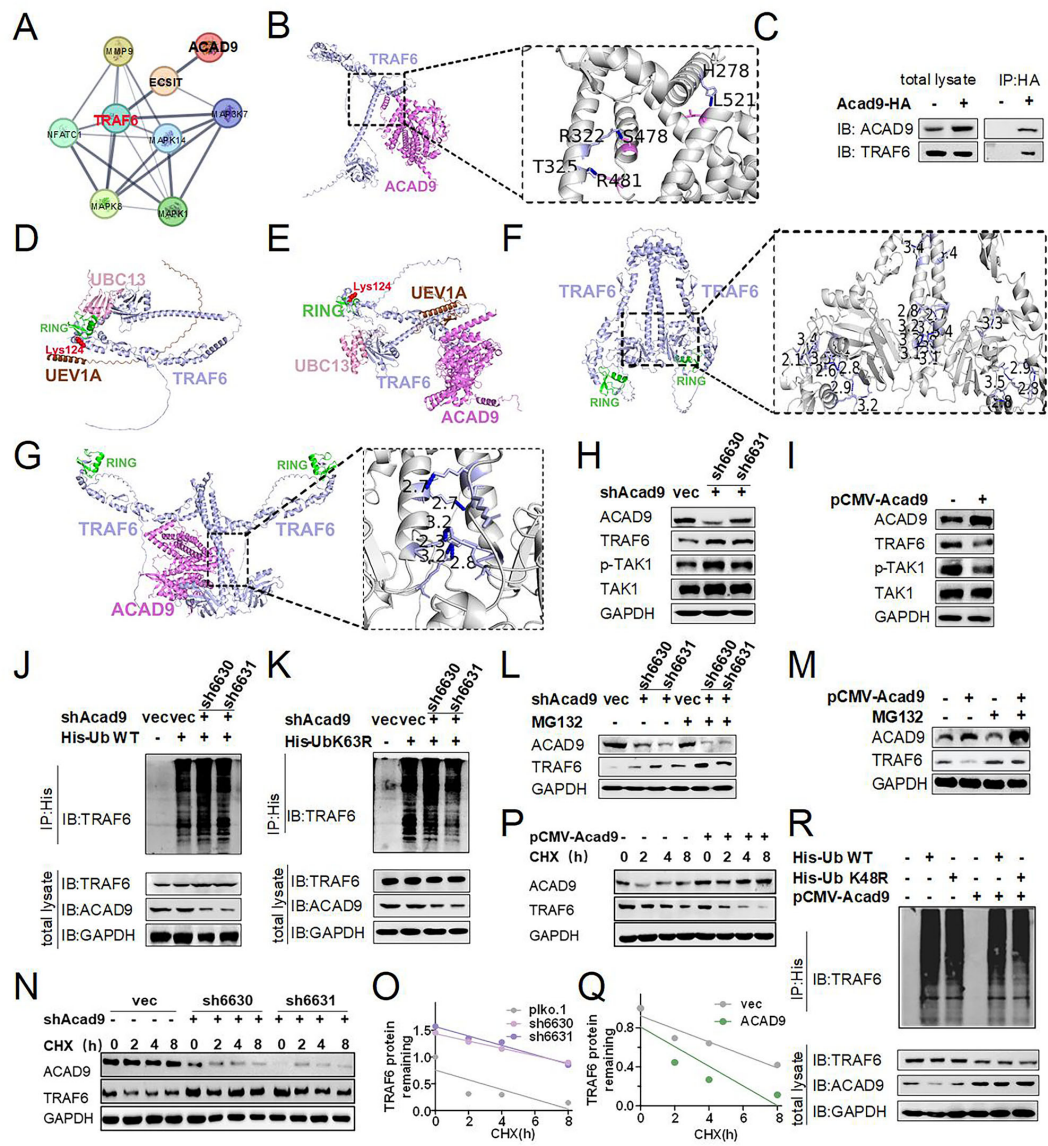
ACAD9 inhibits RANKL-induced osteoclast differentiation by regulating TRAF6 ubiquitination, modification, and degradation. **A** STRING network analysis showing interactions among proteins. **B** Aphafold3 predicted interaction between ACAD9 and TRAF6. The amino acids of the binding site are presented as sticks; light blue presents the protein of TRAF6, purple presents the protein of ACAD9, and blue dashed lines represent hydrogen bonds. **C** Co-IP analysis. The predicted interaction of UBC13, UEV1A, TRAF6 (**D**) in the absence of ACAD9, or **E** in the presence of ACAD9 were shown: TRAF6(light blue), ACAD9(purple), UBC13(pink), UEV1A(brown), RING figure structure(green), the ubiquitination site Lys124(red cartoon). The predicted structure of TRAF6 oligomerization (**F**) in the absence of ACAD9, or (**G**) in the presence of ACAD9, was shown. The number presents the distance between hydrogen and the unit of a hydrogen bond is Angstrom. **H**, **I** The protein expression levels downstream of TRAF6 were detected by western blotting in the indicated RAW264.7 cells. RAW264.7 cells that stably knockdown *Acad9* were transfected with (**J**) His -Ub WT or (**K**) His-Ub K63R plasmids after 48 h, and then analyzed by co-IP. **L**, **M** The protein expression levels of TRAF6 were analyzed in stably knockdown or overexpressed *Acad9* cells with MG132 treatments (10 μg/ml) for 12 h, respectively. **N**, **O**, **P**, **Q** The protein expression levels of TRAF6 were analyzed in stably knockdown or overexpressed *Acad9* cells with CHX treatments (50 μg/ml) for 0, 2, 4, 8 h, respectively. **R** RAW264.7 cells that overexpressed *Acad9* were co-transfected with His-Ub or His-Ub K48R plasmids and then analyzed the interaction of ACAD9 and TRAF6 by co-immunoprecipitation. For in vitro experiments, data represent at least three independent experiments.

The above results highlighted the importance of ACAD9 in regulating TRAF6-mediated signaling, promoting us to further explore the potential molecular mechanism involved. We performed a protein interaction network analysis using STRING, revealing that ACAD9 interacts with ECSIT, and ECSIT interacts with TRAF6 (Fig. 5A). Then, we predicted that ACAD9 interacted with TRAF6 by AlphaFold3(Fig. 5B). Subsequently, we conducted a co-immunoprecipitation experiment in cells, validating the interaction between ACAD9 and TRAF6 (Fig. 5C). Studies have shown that the N-terminal RING and ZnF1 domains of TRAF6 are essential units for catalyzing Lys63-linked polyubiquitin chains. Upon binding to these domains, UBC13/UEV1A transfers ubiquitin to the ubiquitination site of TRAF6. Following this, UBC13/UEV1A dissociates from the RING domain and undergoes multiple rounds of ubiquitin transfer facilitated by E1-Ub, ultimately resulting in the formation of polyubiquitin chains [25, 26]. Additionally, studies have shown that the oligomerization of TRAF6, mediated by its CC domain, is crucial for its self-ubiquitination and downstream signal activation. Oligomerized TRAF6 provides multiple RING domains that can bind multiple UBC13/UEV1A-ubiquitin complexes simultaneously, functioning as a single entity [32]. We speculated that the binding of ACAD9 to TRAF6 could influence the interaction with UBC13, UEV1A, and the RING domains of TRAF6, as well as the oligomerization of TRAF6, potentially inhibiting TRAF6 signal transduction. First, we predicted that ubiquitin-conjugating enzyme (E2) UBC13 and UEV1A bind with the RING and ZnF1 domains of TRAF6 according to the Alphfold3 (Fig. 5D), which is consistent with that of previous studies [33]. However, according to AlphaFold3, when ACAD9 binds to TRAF6, the UBC13 binding site is located on the C-terminal end of TRAF6 rather than on the RING structure, and UEV1A binds to the hyperbolic helix structure of TRAF6 instead of the ZnF1 domain (Fig. 5E), and this binding pattern reduces the intermolecular hydrogen bond interactions that are crucial for TRAF6 oligomerization. Specifically, the number of hydrogen bonds of amino acid binding sites involved in TRAF6 oligomerization decreases significantly from 23 to 6, thereby weakening the intermolecular interaction force (Fig. 5F, G). This conformational change may impair the ability of Lys at the K63 position of TRAF6 to form polyubiquitin chains. Subsequently, we evaluated the activation status of TAK1, a downstream target of TRAF6, following ubiquitination at the K63 site. Consistently, protein levels of TRAF6 and phosphorylation levels of TAK1 were significantly increased in knockdown *Acad9* cells, whereas they were notably reduced in overexpressing *Acad9* cells (Fig. 5H, I). Based on these, we further infer that ACAD9 may have a regulatory effect on the ubiquitination of TRAF6 at K63. Further, we transfected plasmids of His-Ub WT or K63R (only the K63 lysine mutated to arginine) into stable knockdown *Acad9* cells and performed co-IP experiments. The results showed a significant increase in total ubiquitination levels of TRAF6 in knockdown *Acad9* cells (Fig. 5J). Notably, the protein levels of TRAF6 were reduced when we transfected the His-K63R plasmids into knockdown *Acad9* cells, due to the failure of TRAF6 to form a multi-ubiquitin chain at the mutated K63 site (Fig. 5K). These findings suggest that the reduction of ACAD9 primarily led to an increase in the K63-linked ubiquitination of TRAF6.

Studies have demonstrated that TRAF6 not only forms a homodimer to catalyze K63-linked ubiquitination on itself to serve as an anchoring platform for triggering downstream signaling activation but also can be modified by K48-linked ubiquitination and then be degraded by the proteasome to block signaling transduction [26]. Therefore, we speculated that ACAD9 may also influence the degradation of TRAF6 by affecting the status of K48-linked ubiquitination of TRAF6. Firstly, we used proteasomal inhibitor MG132 to inhibit proteasome-dependent degradation and found that the accumulation of TRAF6 increased both under knockdown or overexpressed *Acad9* (Fig. 5L, M). Secondly, we used protein synthesis inhibitor cycloheximide (CHX) to assess the degradation kinetics of TRAF6 and found that the knockdown of *Acad9* delayed the degradation rate of TRAF6, whereas overexpression of *Acad9* accelerated the degradation rate of TRAF6 (Fig. 5N–Q). These results suggest that ACAD9 facilitates TRAF6 degradation via the proteasome system. To further verify this result, we transfected plasmids of His-Ub WT or K48R (only the K48 lysine mutated to arginine) into RAW264.7 cells either overexpressing *Acad9* or not. We found a significant decrease in the protein levels of TRAF6 when the K48 residue of the ubiquitin chain was mutated in cells overexpressing *Acad9* (Fig. 5R).

Collectively, these results strongly suggest that ACAD9 inhibits NF-κB/MAPK signaling pathways by blocking the K63 ubiquitination of TRAF6 and accelerates the degradation of TRAF6 by promoting the K48 ubiquitination of TRAF6.

### Loss of *Acad9* in osteoclast precursors provokes osteoporosis in mice

**Fig. 6:**
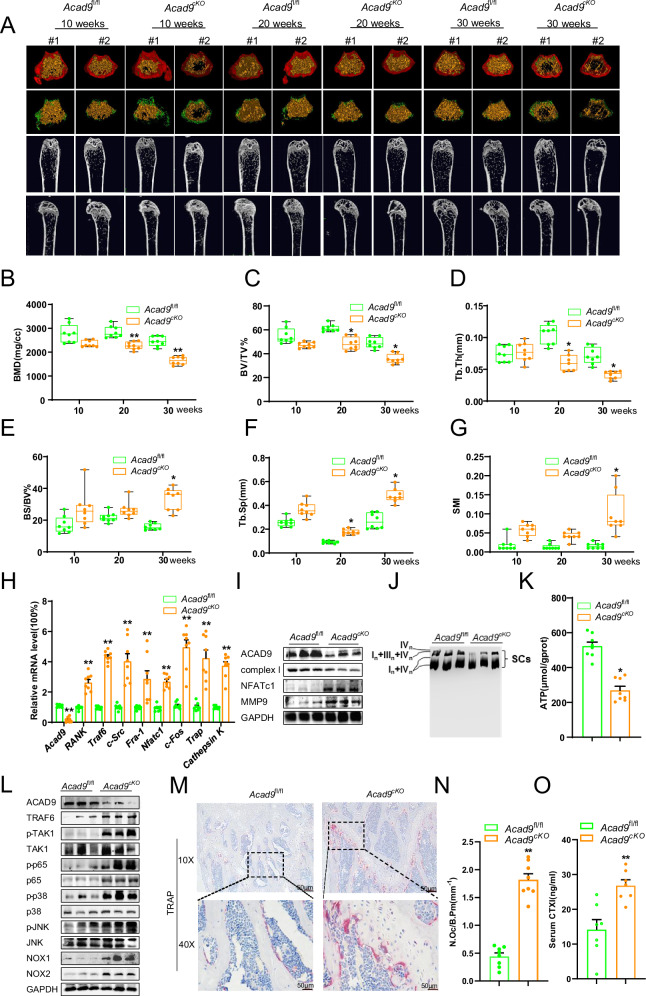
Loss of *Acad9* in osteoclast precursors provokes osteoporosis in mice. Micro-CT images of the femurs of *Acad9*^*fl/fl*^ and *Acad9*^*cKO*^ female mice over time (*n* = 8 mice per group at each time point). **A** The trabecular bone and architecture analyzed by MicroView v2.1.1 Software, (**B**) bone mineral density (BMD), (**C**) bone volume per tissue volume (BV/TV), (**D**) trabecular thickness (Tb. Th), (**E**) bone surface to bone volume (BS/BV), (**F**) trabecular spacing (Tb. Sp), and (**G**) structure model index (SMI). **H**–**L** Samples were from the tibia of *Acad9*^*fl/fl*^ and *Acad9*^*cKO*^ female mice at 30 weeks (*n* = 8 mice per group at each time point): **H** RT-PCR and (**I**) Western blotting analysis of the expression levels of ACAD9, complex I, and markers for osteoclast differentiation. **J** SCs were detected by BN-PAGE. **K** ATP content was detected by the ATP assay kit. **L** The protein expression levels of TRAF6 signaling were detected by western blotting. **M** TRAP staining and (**N**) quantification of osteoclasts of the femurs of *Acad9*^*fl/fl*^ and *Acad9*^*cKO*^ female mice at 30 weeks (*n* = 8 mice per group). **O** The serum CTX-I content was detected by an ELISA kit (*n* = 7 mice per group, 30-week-old mice). Data are shown as Mean ± SEM, *p < 0.05, **p < 0.01 vs *Acad9*^*fl/fl*,^ mice.

To further confirm the essential roles of ACAD9 in osteoclast differentiation and bone resorption in vivo, we generated osteoclast-specific *Acad9*-knockout mice by crossing macrophage-specific Lyz2-cre mice with floxed *Acad9* mice. We evaluated the bone mass by μCT both in female and male *Acad9*^*fl/fl*^, Lyz2-cre (*Acad9*^*cKO*^) mice. The data showed that female *Acad9*^*cKO*^ mice experienced a gradual decrease in bone mass starting at 10 weeks of age, with bone mass continuing to decrease progressively over subsequent months (Fig. 6A, B). The bone volume per tissue volume (BV/TV, Fig. 6C) and trabecular thickness (Tb. Th, Fig. 6D) decreased progressively; the specific bone surface (BS/BV, Fig. 6E) and trabecular separation (Tb. Sp, Fig. 6F) increased progressively in *Acad9*^*cKO*^ mice compared to control mice. Additionally, the structure model index (SMI, Fig. 6G) demonstrated an increasing trend in *Acad9*^*cKO*^ mice, suggesting a shift from rod-like to plate-like bone structures over time, indicative of declining bone mechanical competence. Similar findings were also observed in male *Acad9*^*cKO*^ mice (Supplementary Fig. S3).

We further confirmed the findings in animal tissues, specifically in tibia bone tissue from 30-week-old female mice, which corroborated our observations in cells. Analysis revealed significantly elevated expression of osteoclast regulatory factors (*RANK*, *Traf6*, *c-Src*, and *Fra-1*) and transcription factors (*Nfatc1 and c-Fos*) in *Acad9*^*cKO*^ mice. Additionally, bone resorption markers (*Trap, Cathepsin K*, and MMP9) were increased, while mitochondrial complex I was markedly decreased (Fig. 6H, I). Moreover, we assessed the expression of SCs and measured ATP levels, revealing a significant decrease in the expression of SCs accompanied by reduced levels of ATP in *Acad9*^*cKO*^ mice (Fig. 6J, K). Then, we investigated the status of TRAF6/TAK1/NF-κB/MAPK signaling. We found that phosphorylation levels of TAK1, p65, p38, and JNK were significantly elevated, and the protein expression levels of NOX1/2 were markedly increased in *Acad9*^*cKO*^ mice (Fig. 6L). To assess potential sex-specific effects, we conducted interaction analyses using a general linear model (GLM). Both sexes exhibited similar phenotypes, with no significant sex-by-genotype interactions detected for major bone parameters (BS/TV, BV/TV, Tb.Th, Tb.Sp, and BMD; *p* > 0.05 for all comparisons), indicating consistent phenotypes across both sexes.

To further validate the enhanced osteoclast activity in *Acad9*^*cKO*^ mice, we performed TRAP staining and measured serum bone turnover markers. TRAP staining revealed a significant increase in the number of mature osteoclasts in *Acad9*^*cKO*^ compared to control mice (Fig. 6M, N). Consistent with the histological findings, serum levels of C-terminal telopeptide of type I collagen (CTX-I), a specific marker of bone resorption, were significantly elevated in *Acad9*^*cKO*^ mice (Fig. 6O). These results collectively demonstrate that ACAD9 deficiency in osteoclast precursors promotes osteoclast differentiation and enhances bone resorption without affecting bone formation, thereby contributing to the development of osteoporosis.

These results collectively suggest that ACAD9 deficiency in osteoclast precursors promotes the differentiation of osteoclasts, thereby enhancing bone resorption and contributing to the development of osteoporosis.

## Discussion

Osteoclasts are the sole bone-resorbing cells, and their overactivation disrupts bone homeostasis, particularly in aging populations [5]. RANKL-RANK signaling drives osteoclast differentiation through ROS production and NF-κB/MAPK pathway activation [4, 6]. Targeting these mechanisms—either by reducing ROS or inhibiting NF-κB/MAPK—represent an effective strategy to suppress excessive osteoclast activity and prevent bone loss in osteoporosis.

ROS, generated by NADPH oxidases and mitochondrial oxidases [9, 34, 35], plays a role in osteoclast differentiation. While TRAF6-mediated NOX1/2 activation was considered the primary ROS source [6, 36], mitochondrial complex I serves as the major ROS generator through its structural regulation [34, 37, 38]. ACAD9, a flavoenzyme involved in fatty acid oxidation and complex I assembly [20, 39], was significantly downregulated during osteoclast differentiation. ACAD9 deficiency increased ROS accumulation and activated NF-κB/MAPK signaling, accelerating osteoclastogenesis. Our findings identify ACAD9 as a key regulator of osteoclast differentiation through mitochondrial ROS control.

ACAD9, functioning in mitochondrial complex I assembly [19], serves as a crucial regulator of osteoclastogenesis through dual mechanisms. We show that ACAD9 deficiency reduces complex I levels and ATP production, whereas ACAD9 overexpression prevents RANKL-induced complex I loss and maintains ATP levels. These findings corroborate previous observations that low ATP levels unexpectedly promote osteoclast activity [18, 40]. Importantly, we newly demonstrate that ACAD9 promotes the formation of SCs, enhancing electron transport efficiency to boost ATP synthesis while reducing ROS generation. Beyond these metabolic functions, ACAD9 exhibits significant regulatory effects on osteoclast signaling pathways. Although RANKL activates osteoclastogenesis through the established TRAF6-NOX1/2-MAPK/PI3K/NF-κB cascade [41, 42], NAC-mediated ROS scavenging only partially inhibited differentiation in ACAD9-deficient cells. The opposing effects of ACAD9 knockdown (enhanced activation) and overexpression (reduced activation) on MAPK and NF-κB pathways suggest that ACAD9 directly regulates TRAF6 activity independent of ROS modulation. This reveals an unexpected connection between mitochondrial function and inflammatory signaling in osteoclasts, with ACAD9 serving as a crucial regulatory node.

TRAF6, a member of the tumor necrosis factor receptor-associated factor (TRAF) family, is an important adapter protein that regulates various downstream signaling pathways. It is widely distributed in mammalian tissues and exhibits a highly conserved structure, mainly containing a RING-finger domain in the N-terminal, followed by five Zn-finger domains and a C-terminal TRAF domain [27, 43]. In this study, we observed a negative correlation between the protein expression levels of ACAD9 and TRAF6. Studies indicate that ACAD9 binds to the C-terminal domain of ECSIT, a cytoplasmic protein that interacts specifically with the multi-adaptor protein and E3 ubiquitin ligase TRAF6 [19]. Therefore, we predicted that ACAD9 can bind to TRAF6 using AlphFold3. Furthermore, we confirmed this binding by an immunoprecipitation assay. As a crucial mediator, TRAF6 activated TAK1 primarily as an E3 ubiquitin ligase. This activation occurs through oligomerization via its coiled-coil (CC) domains, which are stimulated when RANKL binds to RANK. Subsequently, TRAF6 is associated with E2 ubiquitin complex UEV1A: UBC13, catalyzing K63-linked polyubiquitination of TRAF6 itself and regulating protein functions and signaling transduction [26, 43, 44]. In this study, we found that the levels of K63-linked polyubiquitination of TRAF6 were signifi cantly increased upon stable knockdown of ACAD9. In contrast, it has been reported that TRAF6 can also be modifi ed by K48-linked polyubiquitination, which targets it for proteasomal degradation, for instance via the E3 ligase c-Cbl [45]. Consistent with this, we found that ACAD9 promotes the degradation of TRAF6 through the ubiquitin-proteasome system.

Interestingly, TRAF6 ubiquitination status in osteoclasts is regulated by multiple molecules with distinct mechanisms. p62 promotes TRAF6 K63-linked polyubiquitination and aggregation, facilitating osteoclastogenesis [46], while CYLD removes K63-linked chains through its deubiquitinase activity, acting as a negative regulator [47]. Our data reveal that ACAD9 employs a dual mechanism distinct from these regulators: it sterically interferes with the TRAF6-UBC13/UEV1A interaction to prevent K63-linked polyubiquitination, while simultaneously promoting K48-linked polyubiquitination for proteasomal degradation. The convergence of multiple regulatory mechanisms on TRAF6 underscores its critical role in osteoclast differentiation. It would be interesting to investigate in future studies whether ACAD9 cooperates with or antagonizes p62 and CYLD in regulating osteoclastogenesis under different physiological or pathological conditions. Given that ACAD9 also regulates mitochondrial function and ROS production, it may serve as an integrator coupling cellular metabolic status with RANKL signaling, a role distinguishing it from purely signaling-oriented regulators like p62 and CYLD.

To further elucidate the role of ACAD9 in bone metabolism, we utilized μCT analysis in osteoclast precursor macrophage *Acad9*-specific knockdown mice and observed progressive bone loss over time, indicating a significant impact of the expression level of ACAD9 on bone density and mass. Importantly, ALP staining of bone sections and serum P1NP measurements revealed no significant differences between *Acad9*^*cKO*^ and control mice (data not shown), indicating that the observed bone loss results primarily from enhanced bone resorption rather than impaired bone formation. Additionally, TRAP staining and immunohistochemistry analysis suggested that the absence of ACAD9 in osteoclast precursors accelerates their differentiation into mature osteoclasts.

An important consideration is that Lyz2-Cre (also known as LysM-Cre) targets myeloid lineage cells, including macrophages and osteoclast precursors, and represents the most widely used mouse line for targeting early stages of the osteoclastic lineage [48]. However, inflammation-mediated bone loss in conditions such as rheumatoid arthritis typically suppresses bone formation while promoting resorption [49, 50], whereas *Acad9*^*cKO*^ mice maintained normal bone formation despite increased resorption. This uncoupled phenotype, together with the successful use of Lyz2-Cre in osteoclast studies [48, 51], supports osteoclasts as the primary cellular target of ACAD9 in bone homeostasis.

ACAD9 mutations cause severe mitochondrial complex I deficiency in humans, presenting with cardiomyopathy, lactic acidosis, and muscle weakness [20], but skeletal health remains unexamined in these patients. Our findings show osteoclast-specific ACAD9 deletion enhances bone resorption and causes osteoporosis in mice through TRAF6-NFATc1 signaling, while human genetic analysis links ACAD9 pLoF variants to increased fracture risk in the UK Biobank. These mechanisms are likely conserved in human osteoclasts, given the essential role of mitochondrial metabolism in osteoclast function, the evolutionarily conserved RANKL-TRAF6 pathway, and the TRAF6-binding motif in ACAD9. Although these variants are ultra-rare (MAF ~ 10⁻⁶–10⁻⁵), the gene-level association and consistent directionality across independent pLoF variants provide robust human genetic evidence. These findings suggest ACAD9 variant carriers may benefit from osteoporosis screening, and future clinical studies should assess bone health in ACAD9-deficient patients. Strategies enhancing ACAD9 activity could offer novel therapeutic approaches for pathological bone loss.

In summary, our study reveals ACAD9 as a dual-functional regulator of osteoclastogenesis with important therapeutic implications. We found that ACAD9 regulates osteoclastogenesis through two distinct mechanisms: maintaining mitochondrial redox balance via complex I/SCs assembly, and inhibiting the TAK1/NF-κB/MAPK axis through a novel interaction with TRAF6. The ACAD9-TRAF6 interaction exerts two critical regulatory effects: allosteric inhibition of TAK1 phosphorylation and promotion of TRAF6 ubiquitination and proteasomal degradation. Our findings suggest that pharmacological enhancement of ACAD9 activity or its TRAF6-binding capacity could provide a novel dual-mechanism approach for preventing pathological bone loss, potentially offering greater efficacy and fewer side effects than current single-target osteoporosis therapies.

## Materials and methods

### Regents and antibodies

Cell culture medium αMEM was purchased from Gibco, and fetal bovine serum was purchased from BI. Recombinant mouse soluble RANKL was purchased from PERPO TECH#315-11C, N-acetyl-L-cysteine(NAC) was purchased from Sigma #A7250, Antibodies against p-P65(ser536) (#3033), p65 (#8242) purchased from Cell Signaling Technology (Beverly, MA), p-P38(Thr180/Tyr182)(sc-166182), p38(sc-7972), p-JNK(Thr183/Tyr185) (sc-7345), JNK(sc-6254) were purchased from Santa Cruz Biotechnology (Santa Cruz, CA), ACAD9(ab220386) and NOX1(131008) antibodies were purchased from Abcam (Abcame Cambridge, UK). Antibodies NFATc1(A19597), NOX2(19701), TAK1(A19077), p-TAK1(Tyr187) (AP1222), and TRAF6(A23385) were purchased from ABclonal (Wuhan, China). Antibodies against mitochondrial complexes I (39 kDa, #459130), II (30 kDa, #459230), III (51 kDa, #459140), IV (40 kDa, #459600), and V (55 kDa, #459240) were purchased from Invitrogen (Carlsbad, CA). TRAP staining kit (Acid Phosphatase, Leukocyte TRAP kit) was purchased from Sigma (#387A). ATP assay kit (#A095-1-1) was purchased from Nanjing Jiancheng Bioengineering Institute (Nanjing, China). H_2_DCF-DA (#D399) was purchased from Life Technologies (San Diego, CA). Anti-Flag® M2 affinity Gel was purchased from Sigma(#A2220).

### Plasmids and lentivirus production

Overexpressed *Acad9* plasmid was obtained from Sino Biological (#MG53598-CY), containing ACAD9 cDNA ORF Clone for mice with a C-terminal HA tag. The overexpression *Traf6* plasmid was purchased from MiaoLing Biology (#P19727), designated as pcDNA3.1-TRAF6. Short hairpin RNAs (shRNAs) targeting *Acad9* were synthesized and cloned into the pLKO.1-puro vector for knockdown experiments. The specific targeting sequences for *Acad9* were 5′-GCCCGCATTCTCCTAATCTTT-3′ and 5′-CCTCATCAACTTGTATGGCAT-3′. A pLKO.1-puro vector containing a non-targeting scramble sequence (pLKO.1-empty) was used as a control. Lentivirus was produced by transfecting 293FT cells with the respective expression plasmids along with packaging plasmids pCMV-dR8.9 and pCMV-VSV-G. After 48 h, lentivirus containing shRNA or empty vectors was used to infect RAW264.7 cells for 48 h. Subsequently, infected cells were selected with puromycin in the conditioned medium for 7–10 days.

### Cell culture and treatments

The mouse leukemic monocyte/macrophage RAW264.7 cell line was purchased from ATCC and cultured in αMEM supplemented with 10%(v/v) heat-inactivated FBS at 37 °C in a humidified 5% CO_2_ atmosphere. For in vitro osteoclastogenesis, cells were seeded in 12-well plates at a density of 1 × 10^4,^ and the medium was supplemented with 100 ng/ml RANKL for differentiation under cells reached confluence. The differentiation medium was changed every 2 days, followed by conducting other detection experiments. To assess the differentiation of RAW264.7 cells at the end of the culture period, the medium was discarded, and cells were washed three times with PBS, forwarded by staining for TRAP according to the manufacturer’s instructions of the TRAP stain kit.

### Real-time PCR analysis and Western Blotting analysis

RT-PCR analysis and Western Blotting analysis were performed as described previously [52]. Details regarding the primers used in the RT-PCR can be found in the Supplementary Material.

### *Acad9* conditional KO mice, OVX mice, and in vivo experiments

The Cre/loxP system was utilized to generate tissue-specific transgenic mice. Specifically, Acad9-floxed mice were generated by GemPharmatech Co., Ltd (Nanjing, China). These mice, denoted as *Acad9*^*fl/fl*^ for homozygotes and *Acad9*^fl/+^ for heterozygotes, were engineered with two sgRNAs to insert loxP sites along with a 658 bp coding sequence (encompassing exon 2–7) into the *Acad9* locus of the mouse genome. All mice used in the study were of the C57BL/6J background and had been backcrossed for at least seven generations. To achieve macrophage-specific knockout of *Acad9*, the *Acad9*^*fl/fl*^ mice were crossed with B6-Lyz2-cre mice (strain No T003822, GemPharmatech Co. Ltd, Nanjing, China). Lyz2-Cre mice were crossed with *Acad9*^*fl/fl*^ mice to generate osteoclast precursor macrophage-specific knockout mice: homozygous (*Acad9*^*fl/fl*^, Lyz2-Cre, labeled as *Acad9*^*cKO*^) and heterozygous (*Acad9*^fl/+^, Lyz2-Cre). Littermates with the *Acad9*^*fl/fl*^ genotype served as controls. All animals were housed in a controlled environment at 25 °C, 60% humidity, under a 12-h light/12-h dark cycle, and fed a standard chow diet.

For ovariectomized (OVX) mice, 8-week-old female C57BL/6 mice were acquired from the Experimental Animal Center of Xi’an Jiaotong University for this study. At the beginning of the experiment, the mice were randomly assigned to two groups, each containing four mice: (1) sham group, mice received operation but without removal of the ovaries; (2) OVX group, the mice received operation of ovariectomy was performed as previously described [53]; After 2 months, all mice were sacrificed, and tissues were collected for analysis. For all animal studies, blinding was strictly enforced. The experimental animal protocol was approved by the Experimental Animal Ethics Committee of Xi’an Jiaotong University.

### Bone structure evaluation with micro-CT

Micro-computed tomography (μCT) analysis of trabecular bone in the femur was performed using a PerkinElmer Quantum GX micro-CT system (CLS149276). Parameters including bone mineral density (BMD), bone surface-to-bone volume ratio (BS/BV), bone volume fraction (BV/TV), trabecular thickness (Tb. Th), trabecular separation (Tb. Sp), and structure model index (SMI) were calculated from the region of interest (ROI). Radiographs were generated with 2-dimensional cross-sections and 3-dimensional reconstructions. All micro-CT data were processed and analyzed using Micro View v2.1.1 software.

### Immunohistochemistry

The tri-formol-fixed femur tissues from WT and OVX were used for immunohistochemistry staining for ACAD9. After dewaxing, the embedded sections were heated with 0.01 M citrate buffer for 10 min for antigen repair, then sealed with 5%BSA and inactivated with hydrogen peroxide, then incubated with 1:100 dilution of ACAD9 antibody at 4 °C for 24 h, rinsed with PBS three times, and incubated with a biotinylated secondary antibody (dilution 1:150) for 1 h, finally 0.05% diaminobenzidine that generated a brown color, Nuclei were presented with hematoxylin staining, and all slides were observed under the optical microscope.

Femur tissues were dissected from *Acad9*^*fl/fl*^ and *Acad9*^*cKO*^ mice were fixed using 10% formalin for 48 h and decalcified in 14% EDTA (pH 7.4) at room temperature. After being frozen, sagittal-oriented sections were prepared for histological analysis and TRAP staining according to the manufacturer’s protocol. TRAP-positive cells with three or more nuclei were counted as osteoclasts under the bright field of a fluorescence microscope.

### Serum CTX-I measurements

Blood samples were collected from 30-week-old mice via retro-orbital bleeding after overnight fasting. Serum was separated by centrifugation (3000 rpm, 15 min, 4 °C) and stored at −80 °C until analysis. Serum levels of CTX-I were measured using commercial ELISA kits specific for mouse samples (#E-EL-M3023, Elabscience, Wuhan, China) according to the manufacturer’s instructions. Briefly, 100 μL of diluted serum samples or standards were added to antibody-coated plates and incubated at 37 °C for 90 min. After washing, biotin-conjugated detection antibody and HRP-streptavidin were sequentially added with 60 min and 30 min incubations, respectively. TMB substrate was added for color development, and absorbance was read at 450 nm.

### Szr4dLoF gene burden associations with fracture

To investigate the association between coding variations in the ACAD9 and fractures, we obtained data on rare predicted loss-of-function (pLoF) gene burden associated with fractures from the Genebass browser. Based on this data, we screened all pLoF genes that showed significant associations (SKAT-O *p* < 0.05) with fractures. A Manhattan plot was then generated to visualize the distribution of pLoF gene variants related to fractures.

### ACAD9 variant associations with fracture

We obtained all single-variant information related to ACAD9 and fractures from the Genebass browser. Based on the beta score and *P* value, we screened for the single variants in ACAD9 that contribute the most to fracture.

### Intracellular ROS assay and ATP content detection assay

ROS levels were assessed using the non-fluorescent probe 2′,7′-dichlorofluorescein diacetate (DCFH-DA), which is converted to the fluorescent compound 2′,7′-dichlorofluorescein (DCF) upon oxidation, following established methods. Briefly, cells were seeded in 12-well plates and treated with RANKL (100 ng/ml) in the presence or absence of NCA for 4 days. After treatment, cells were washed with PBS and incubated with DCFH-DA (10 μM) in the dark at 37 °C for 30 min. Fluorescence intensity was then measured using a fluorescence spectrometer (FlexStation 3, Molecular Devices, Sunnyvale, CA) with excitation at 485 nm and emission detection at 538 nm. Intracellular ROS levels were quantified as relative DCF fluorescence per microgram of protein, determined using the BCA protein assay method. The collection of cell and tissue samples and the detection of ATP content were performed according to the manufacturer’s instructions for the ATP detection assay kit.

### Mitochondrial isolation and Blue Native-PAGE in-gel activity assay

After reaching confluence, cells were harvested and washed with PBS. They were then resuspended in an isolation buffer containing 0.25 M sucrose, 5 mM Tris/HCl (pH 7.4), and 1.5 mM EGTA, with PMSF added immediately before use to inhibit protease activity. The cell suspension was homogenized using a Duane’s glass homogenizer. Then, they were centrifuged at 600 × *g* for 10 min to remove nuclei and large debris. This step could be repeated as necessary to ensure the complete removal of precipitates. Finally, centrifuged at 7000 × *g* for 10 min to pellet the mitochondrial fraction.

For the detection of SCs using Blue Native PAGE, the following steps were performed according to established protocol: approximately 12 μg of protein per lane was loaded onto Blue Native PAGE gels. Electrophoresis was carried out at 150 V for 30 min, followed by 250 V for 45–60 min to separate protein complexes. Separated proteins were transferred onto a PVDF membrane using a standard transfer apparatus at 200 mA for 6 h. Immunodetection of SCs was performed using an antibody specific to NDUFA9 (Invitrogen #459100).

### Protein half-life CHX chasing assay, in vitro ubiquitination assays

To determine the effect of ACAD9 on the half-life of TRAF6, a CHX-based assay was performed. Briefly, we treated RAW264.7 cells, including normal, knockdown *Acad9*, and overexpression *Acad9* cells with CHX (50 μg/ml) over a time course of 0 h up to 8 h, harvested cells and prepared protein samples, and finally analyzed them by western blotting.

The in vitro ubiquitination assays were performed. Briefly, we constructed His-tagged Ubiquitin (His-Ub) and mutant Ubiquitins: His-K63R (mouse Ub, only K63 lysine mutated to arginine), His-K48R (mouse Ub, only K48 lysine mutated to arginine), and then we co-transfected His-Ub or mutant plasmids into RAW264.7 cells, including normal cells and knockdown *Acad9* or overexpression *Acad9* cells. 48 h after transfection, MG132 was added to block proteasome degradation for 8 h, and cells were harvested in denatured buffer. Subsequently, all samples were analyzed by the co-IP assay.

### Co-immunoprecipitation assays

After 48 h of transfection, cells were washed twice with ice-cold PBS and lysed using IP lysis buffer. Subsequently, cell lysates were clarified under centrifugation at 1500 rpm for 10 min at 4 °C. 30 μl of the protein lysate was served as a cell lysate sample for analysis. The remaining protein lysates were incubated with anti-Flag M2 beads or BeyoGold^TM^ His-tag Purification Resin at 4 °C for 4 h. After incubation, samples were centrifuged at 10,000 rpm for 10 min at 4 °C, discarded the supernatant, and collected the precipitate. The collected resin-bound complex was washed four to five times with NETN buffer. Finally, the washed resin-bound complexes were analyzed by Western blotting experiments.

### Gene-Module Association Determination (G-MAD) analysis and functional protein association networks analysis

G-MAD analysis as previously reported. Briefly, transcriptome datasets of bone and bone marrow tissues obtained from large cohorts (datasets with over 40 samples) utilize the PEER-derived expression residuals as Steele et al. report. Functional protein association networks analysis using STRING network (http://www.string-db.org).

### Prediction with AlphaFold3 and visualized by Pymol

All the protein amino acid sequences that need to be predicted are derived from NCBI, and the process of predicting protein interaction structure follows AlphaFold Server (https://golgi.sandbox.google.com/about). In brief, upload the amino acid sequence of the predicted protein on the task submission interface, save and submit, wait for the completion of the task calculation, and then download the result. Finally, use the Pymol software to perform visual analysis on the one with the highest score.

### Statistical analysis

All data are expressed as means ± SEM. Statistical analyses were performed using GraphPad Prism 8 software (San Diego, CA) or SPSS software (IBM SPSS Statistics, R27.0.1.0). Comparison of two independent groups was done with Student’s *t*-test. Sex-specific effects were evaluated using a GLM in SPSS with sex and genotype as fixed factors and bone parameters as dependent variables. Sex-by-genotype interactions were assessed to determine whether phenotypes differed between male and female mice. Statistical significance was set at *p* < 0.05.

## Supplementary information

Moonlighting Cytosolic Function of ACAD9: Suppression of TRAF6-Mediated Osteoclastogenesis and Protection Against Osteoporosis

Uncut gel western blot data

## Data Availability

All cell lines, plasmids, and mice generated for this study are available from the lead contact upon request.

## Code availability

All original code has been deposited at the repository and is publicly available at (DOI) as of publication. This paper does not report the original code.
