# Supplementary material for: Moonlighting cytosolic function of ACAD9: suppression of TRAF6-mediated osteoclastogenesis and protection against osteoporosis
Source: Cell Death Dis. 2026 Mar 26;17(1):362. doi: 10.1038/s41419-026-08626-z (PMC13039524; doi:10.1038/s41419-026-08626-z)
Supplement: Supplementary file 1 — Moonlighting Cytosolic Function of ACAD9: Suppression of TRAF6-Mediated Osteoclastogenesis and Protection Against Osteoporosis [file 41419_2026_8626_MOESM1_ESM.docx]

**Supplemental Materials**

**Moonlighting Cytosolic Function of ACAD9: Suppression of TRAF6-Mediated Osteoclastogenesis and Protection Against Osteoporosis**

Mimi Wang ^a^, Chao Yuan ^a^, Yi Zhang ^a^, MengMeng Peng ^a^, Yundie Liu ^a^, Ruolin Liu ^b^, Zhaode Feng ^a^, Zhiwei Yang ^c^, Hao Li ^a^, Zhongbo Liu ^b^ and Ying Cheng ^a,*^

^a^ Center for Mitochondrial Biology & Medicine, Key Laboratory of Biomedical Information Engineering of Ministry of Education, School of Life Science and Technology, Xi'an Jiaotong University, Xi'an, China.

^b^ Key Laboratory of Shaanxi Province for Craniofacial Precision Medicine Research, Laboratory Center of Stomatology, College of Stomatology, Xi’an Jiaotong University, Xi’an, China.

^c^ MOE Key Laboratory for Nonequilibrium Synthesis and Modulation of Condensed Matter, School of Physics, Xi’an Jiaotong University, Xi'an, China.

* Corresponding author: Ying Cheng, Email: yingcheng@xjtu.edu.cn

**Methods**

**Immunohistochemistry**

The tri-formol-fixed femur tissues from WT and OVX were used for immunohistochemistry staining for ACAD9. After dewaxing, the embedded sections were heated with 0.01M citrate buffer for 10 min for antigen repair, then sealed with 5%BSA and inactivated with hydrogen peroxide, then incubated with 1:100 dilution of ACAD9 antibody at 4℃ for 24h, rinsed with PBS three times, and incubated with a biotinylated secondary antibody (dilution 1:150) for 1 h, finally 0.05% diaminobenzidine that generated a brown color, Nuclei were presented with hematoxylin staining, and all slides were observed under the optical microscope.

**Cell culture and NAC treatments**

For in vitro osteoclastogenesis, seeded cells in 12-well plates and the medium supplemented with 100ng/ml RANKL for differentiation under cells reached confluence, and the differentiation medium was changed every 2 days. For the N-acetyl-L-cysteine (NAC) intervention experiment, during the osteoclastogenesis, pretreated cells with NAC(5μM) or not and conducted other detection experiments.

**Functional enrichment analysis**

The pathway terms categorized by the Kyoto Encyclopedia of Genes and Genomes (KEGG) Pathway Database and Gene Ontology (GO) Process were collected. Osteoporosis-related biological terms were selected, and the matching genes were counted.

**Table S1**


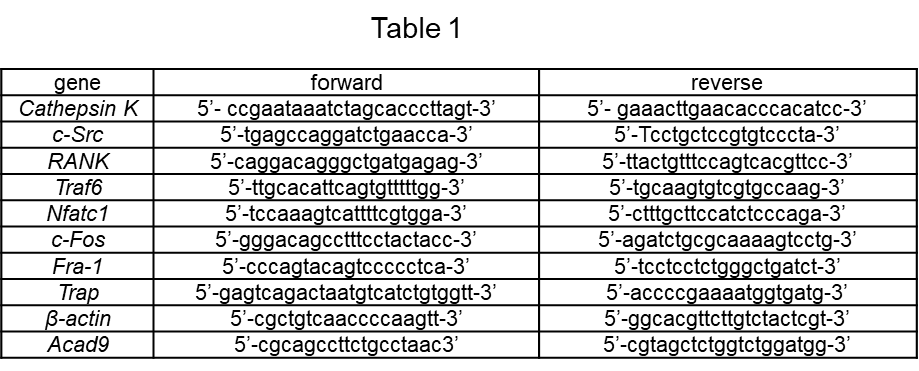


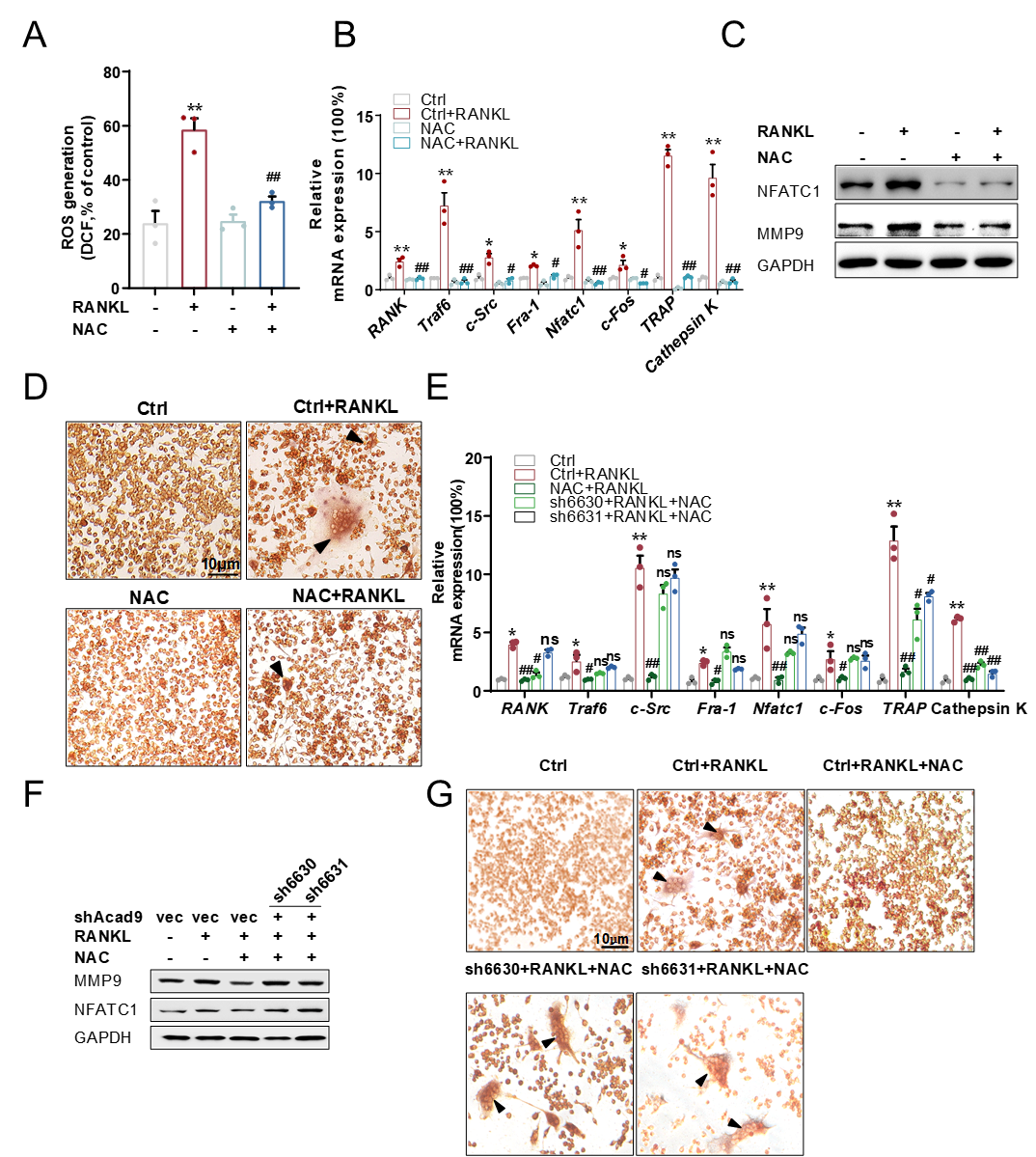


**Fig. S1: Clearing ROS alone does not completely inhibit osteoclast differentiation and maturation due to loss of ACAD9 function.** (A) ROS levels were detected using DCFH2-DA, (B) the relative mRNA levels of RANKL, Traf6, c-src, Fra-1, NFATc1, c-fos, TRAP, and cathepsin K were tested by qRT-PCR, (C) the levels of relative osteoclast differentiation marker protein NFATc1 and MMP9 were detected by western blot and (D) TRAP staining was performed from cells pre-treated NAC or not after induced 4 days.(E) the relative mRNA levels of RANKL, Traf6, c-src, Fra-1, NFATc1, c-fos, TRAP, and cathepsin K were tested by qRT-PCR, (F) the levels of relative osteoclast differentiation marker protein NFATc1 and MMP9 were detected by western blot and (G) TRAP staining was performed from cells that stably knockdown ACAD9 pre-treated with NAC or not after induced 4 days. For *in vitro* experiments, data represent at least three independent experiments. Data are shown as Mean ± SEM, ∗p<0.05, ∗∗ p<0.01 vs. Ctrl group; # p<0.05, ##p<0.01 vs. Ctrl+RANKL group.

**
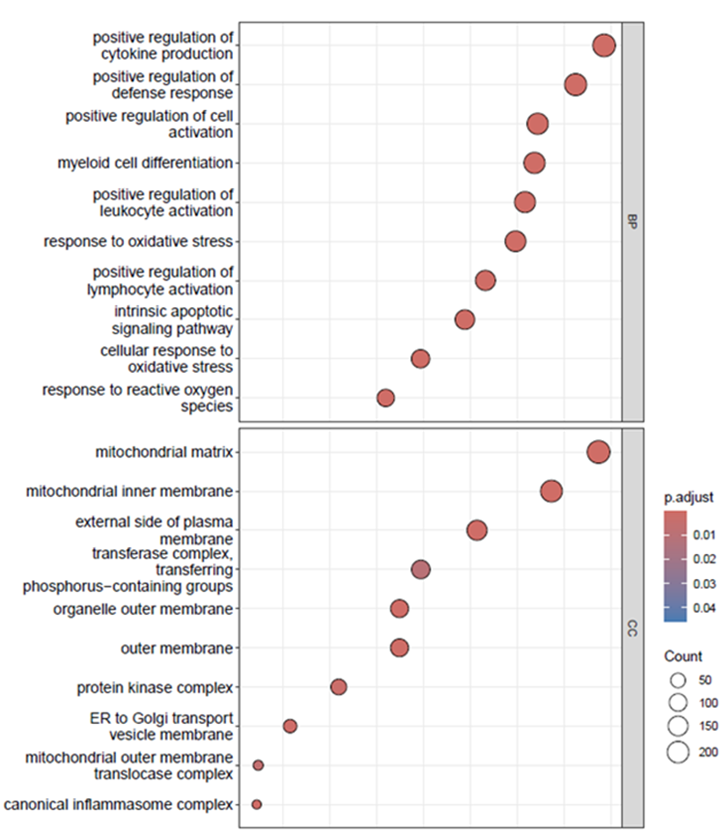
**

**Fig. S2: ACAD9 is related to the composition of the mitochondrial inner membrane.** (A) Gene Ontology (GO) enrichment analyses and functional annotations


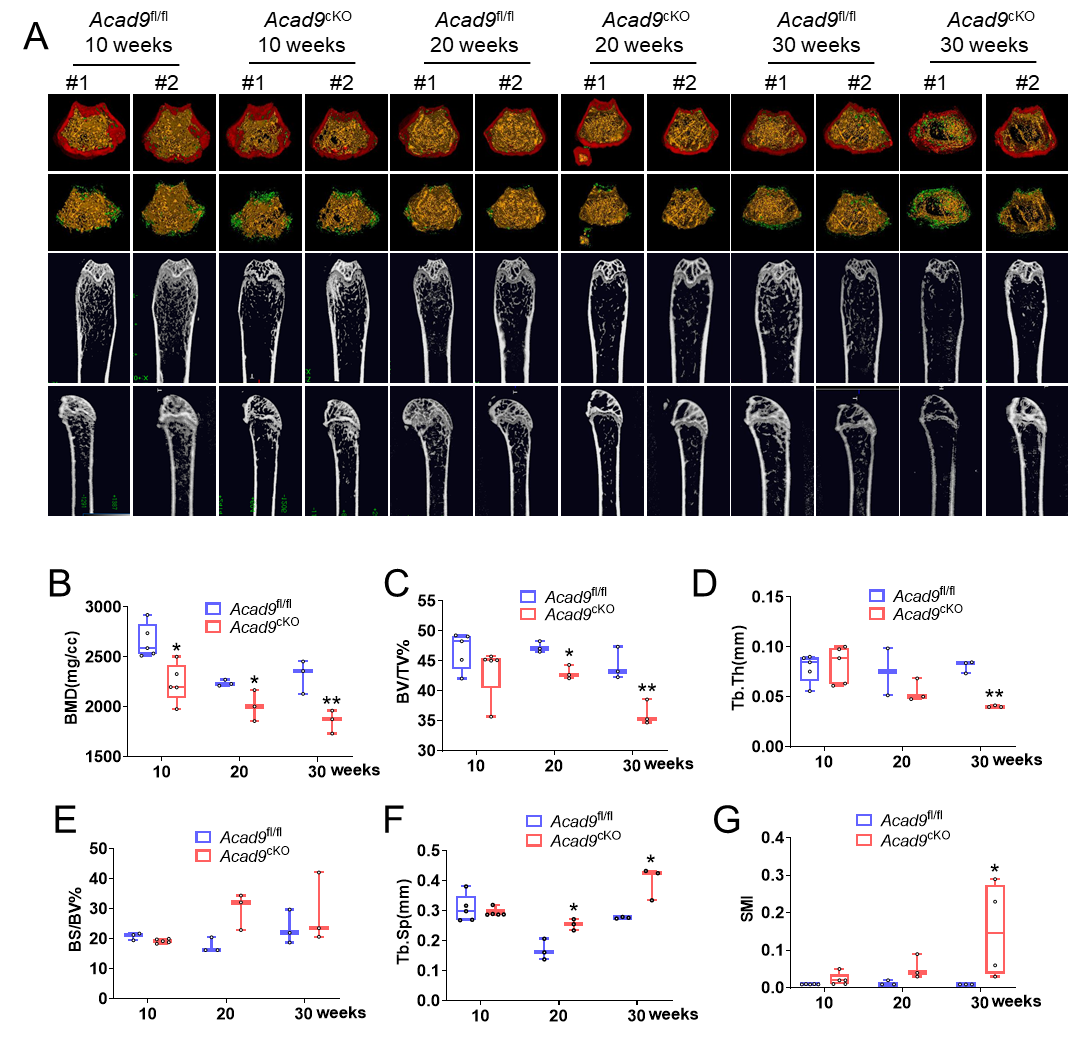


**Fig. S3:** **Osteoclast precursors loss of *Acad9* provoke osteoporosis in male mice**. Micro-CT images of the femurs of *Acad9*^fl/fl^ (n=3-5), and *Acad9*^cKO^ (n=3-5) male mice over time (A). The trabecular bone and architecture analyzed by MicroView v2.1.1 Software: (B) bone mineral density (BMD), (C) bone volume per tissue volume (BV/TV); (D) trabecular thickness (Tb. Th); (E) bone surface to bone volume (BS/BV); (F) trabecular spacing (Tb. Sp); and (G) structure model index (SMI), Data are shown as Mean ± SEM, ∗*p*<0.05, ∗∗ *p*<0.01 vs. *Acad9*^fl/fl^ mice.
