# Supplementary material for: Moonlighting cytosolic function of ACAD9: suppression of TRAF6-mediated osteoclastogenesis and protection against osteoporosis
Source: Cell Death Dis. 2026 Mar 26;17(1):362. doi: 10.1038/s41419-026-08626-z (PMC13039524; doi:10.1038/s41419-026-08626-z)

Figure 1 western blot picture

70KD

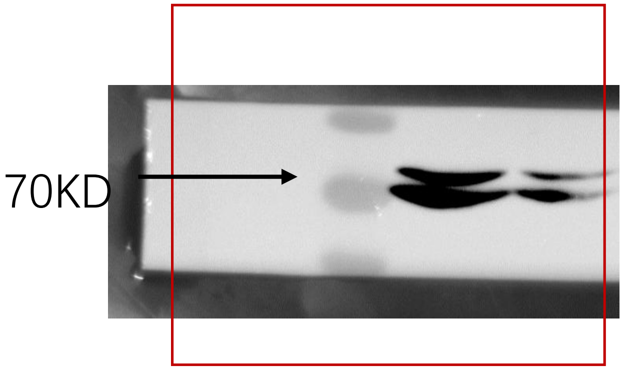

D: ACAD9 69KD

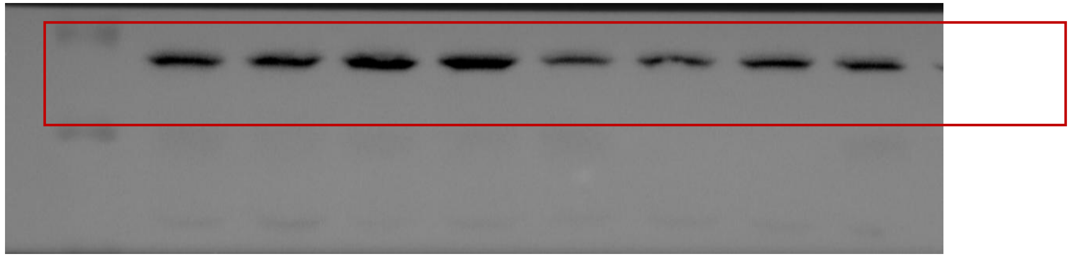

G: ACAD9

Figure 2 western blot picture

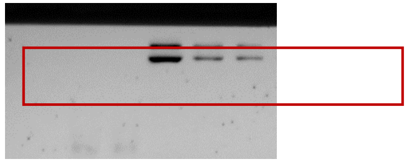

B: ACAD9

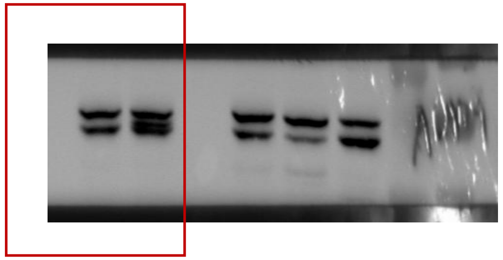

H: ACAD9

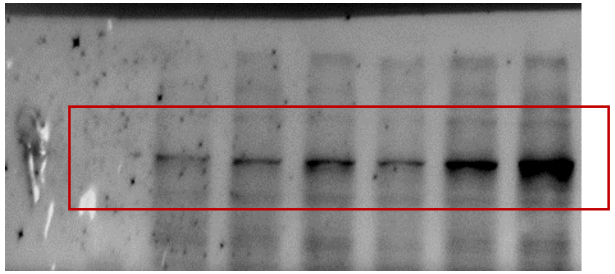

C: MMP9

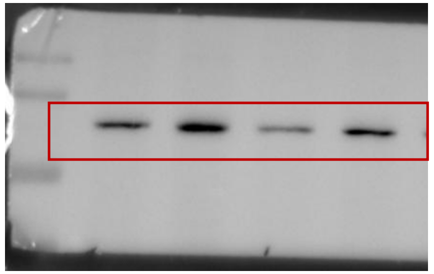

J: NFATc1

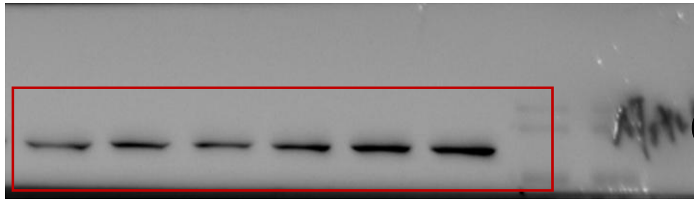

C: NFATC1

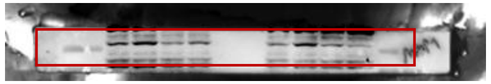

J: NFATc1

Figure 3 western blot picture

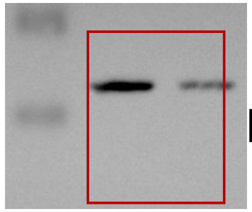
Fig3C: complex II

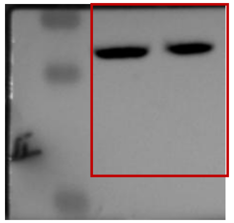
Fig3C: complex II

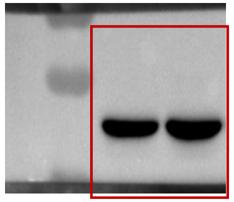
Fig3C: complex III

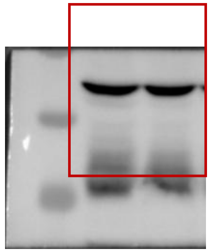
C: complex IV

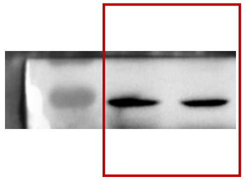
C: complex V

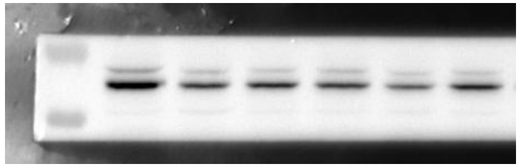
Fig3D: ACAD9

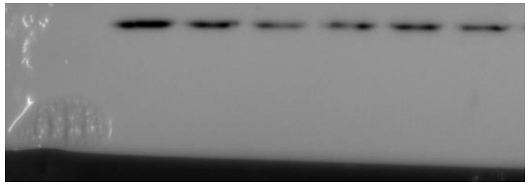
Fig3D: complex I

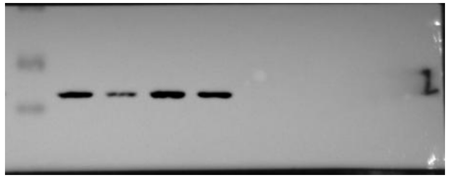
Fig3E: complex II

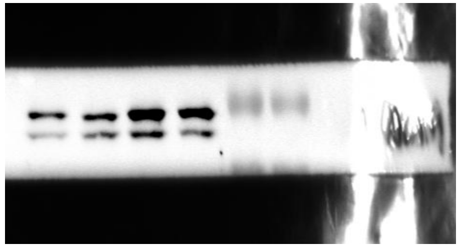
Fig3E: ACAD9

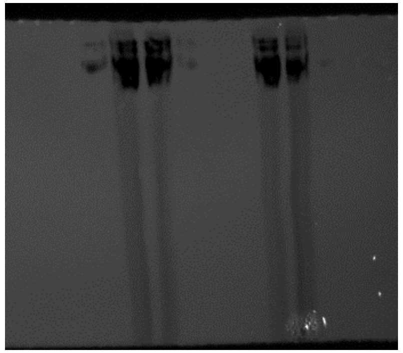
Fig3G: supercomplex

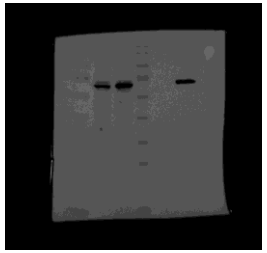
Fig 3K: IP ACAD9

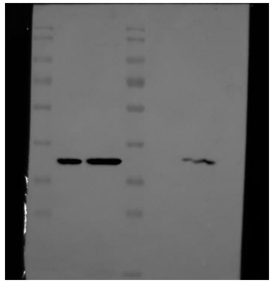
Fig3K: IP complex I

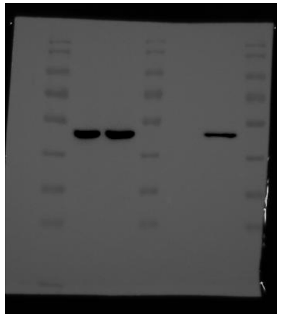
Fig3K: IP complex III

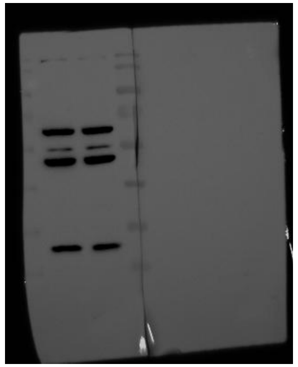
Fig3K: IP complex II

Figure 3 western blot picture

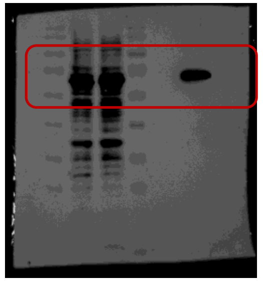

Fig3K: IP complex IV

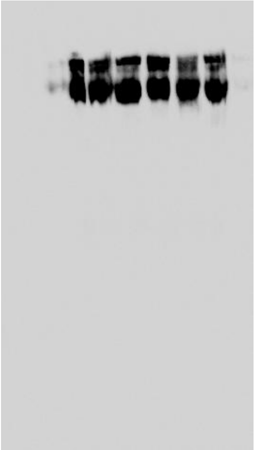

Fig3L: supercomplex

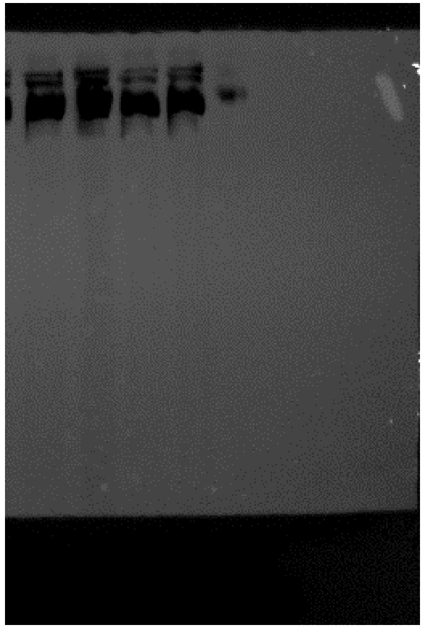

Fig3N: supercomplex

Figure 4 western blot picture

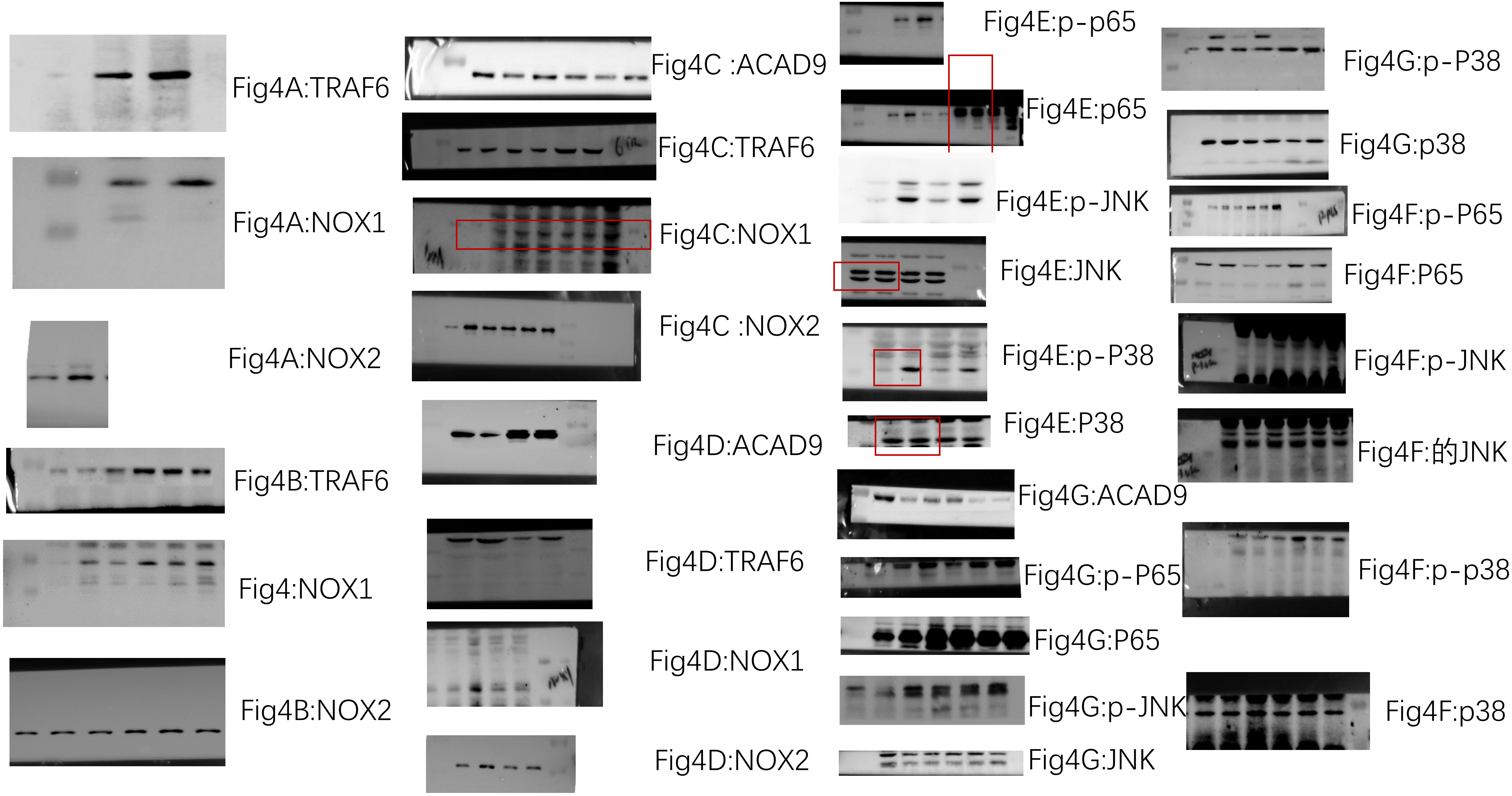

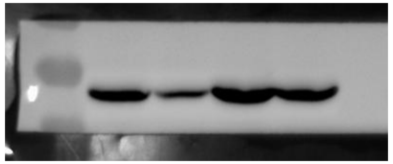

Fig4H:ACAD9

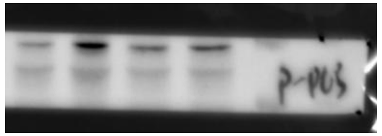

Fig4H:p-p65

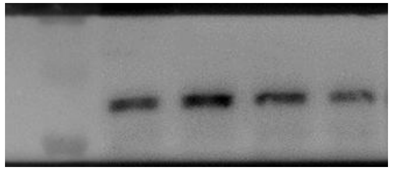

Fig4H:p65

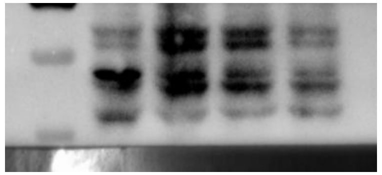

Fig4H:p-JNK

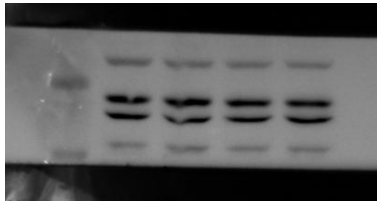

Fig4H:JNK

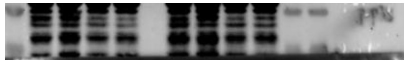

Fig4H:p-p38

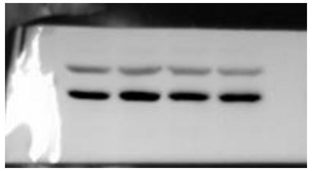

Fig4H:p38

Figure 5 western blot picture

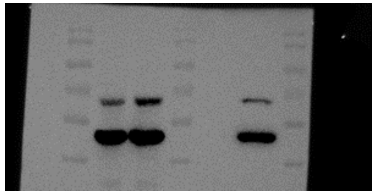
Fig5 C

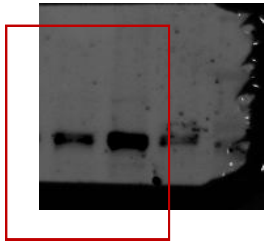
Fig5H:ACAD9

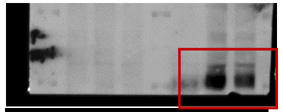
Fig5H:p-TAK1

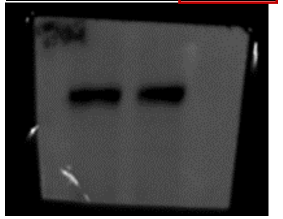
Fig5H:TAK1

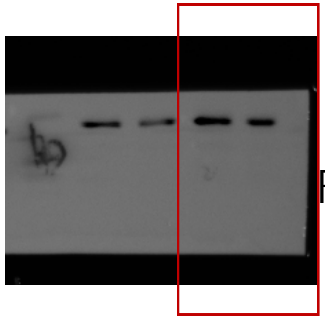
Fig5H:TRAF6

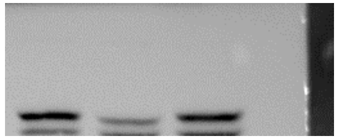
Fig5I:ACAD9

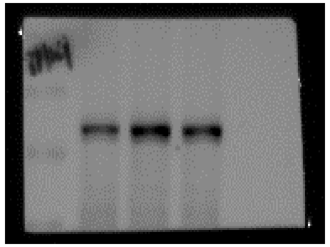
Fig5I:p-TAK1

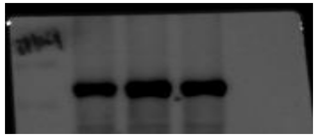
Fig5I:TAK1

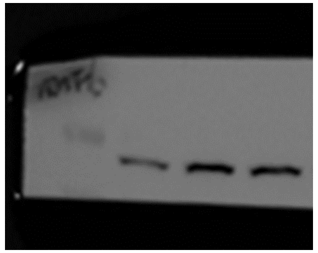
Fig5I:TRAF6

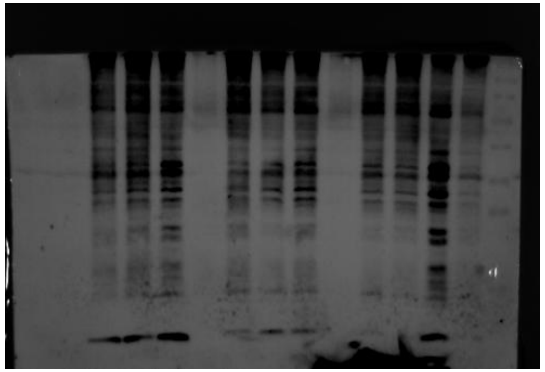
Fig5J IP WB:TRAF6

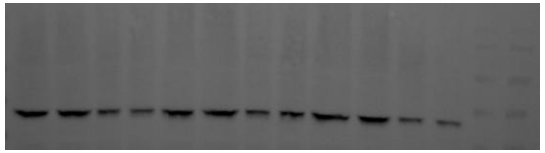
Fig5J:ACAD9

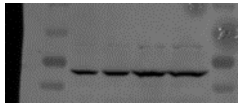
Fig5J: Total lysate TRAF6

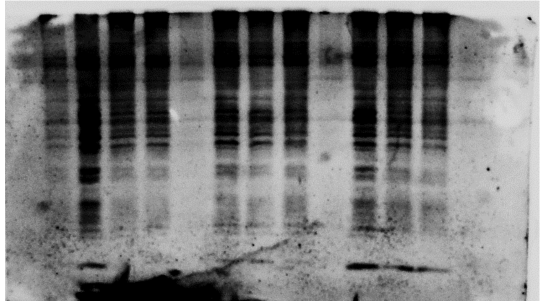
Fig5K IP WB:TRAF6

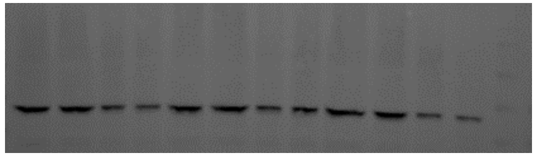
Fig5K:ACAD9

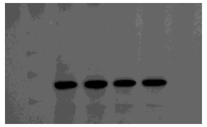
Fig5K: total lysate TRAF6

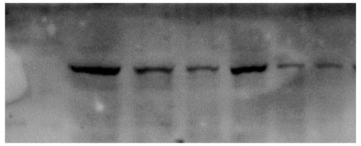

Fig5L:ACAD9

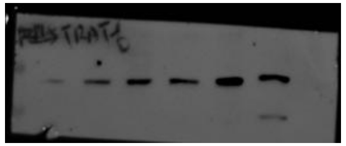

Fig5L:TRAF6

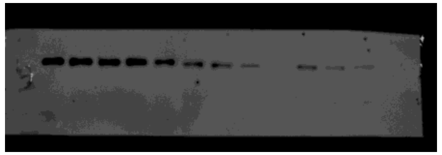

Fig5N:ACAD9

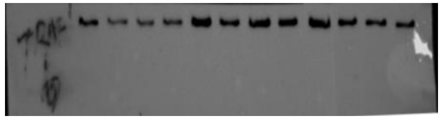

Fig5N:TRAF6

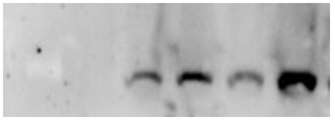

Fig5M:ACAD9

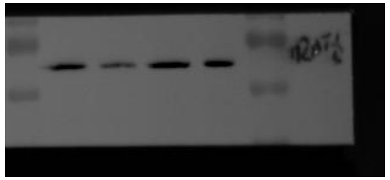

Fig5M:TRAF6

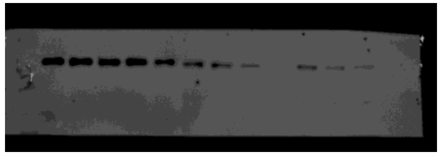

Fig5N:ACAD9

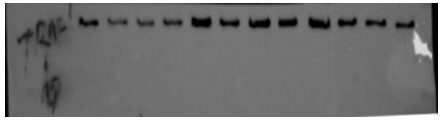

Fig5N:TRAF6

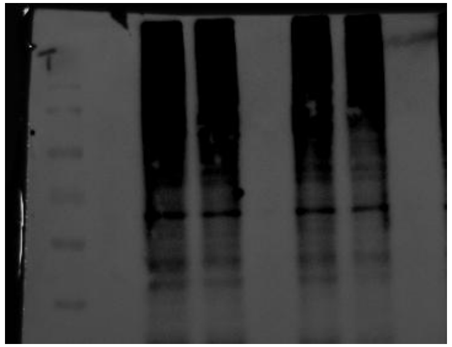

FigR: IP WB:TRAF6

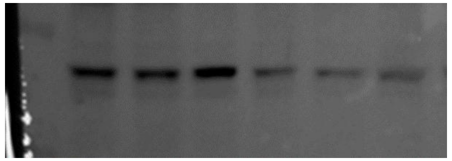

FigR:TRAF6

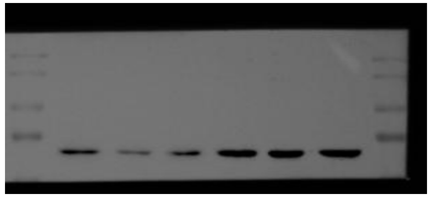

FigR:ACAD9

Figure 6 western blot picture

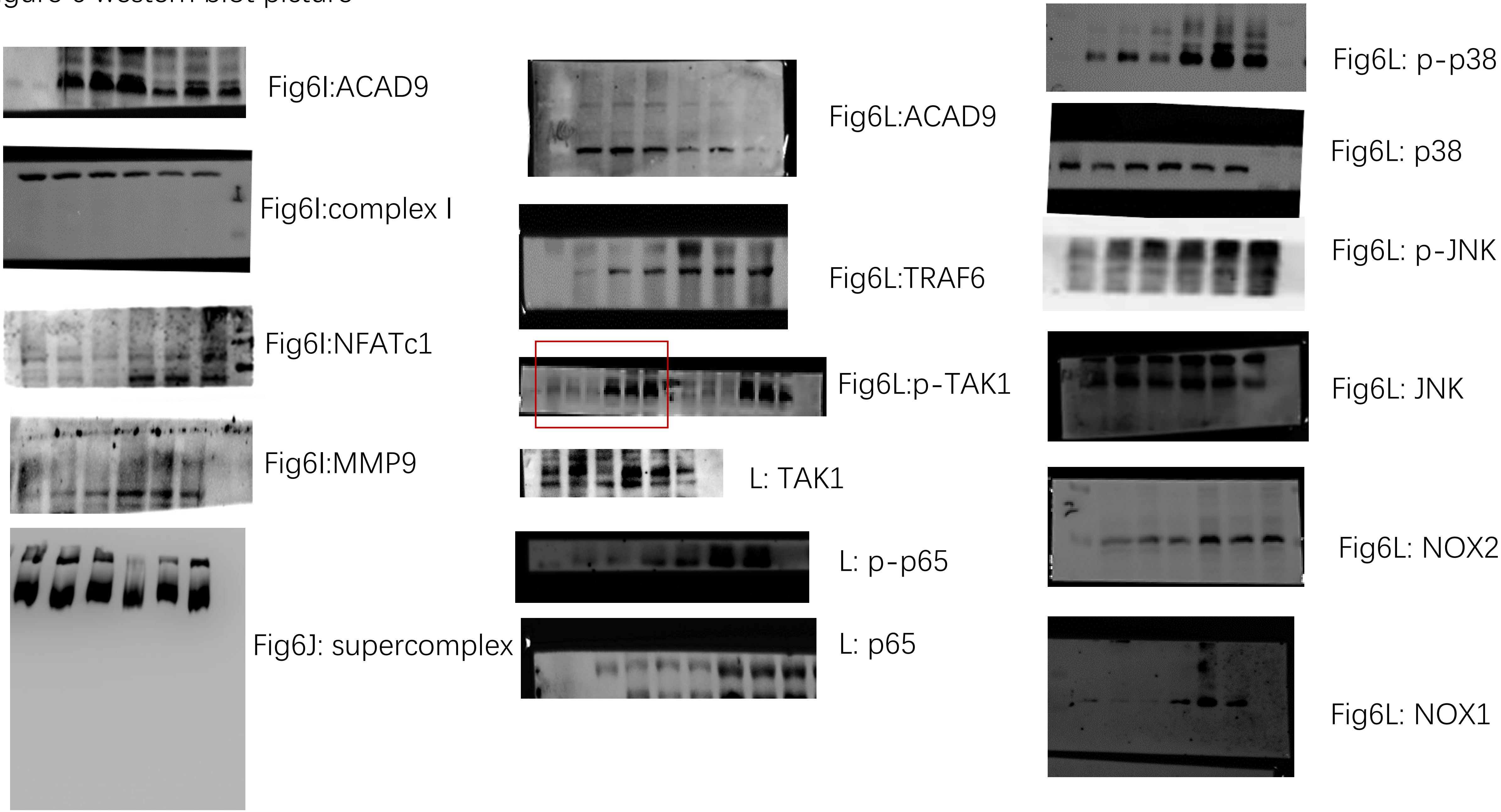

Supplement: Supplementary file 2 — Uncut gel western blot data [file 41419_2026_8626_MOESM2_ESM.pdf]
